# Towards time resolved characterization of electrochemical reactions: electrochemically-induced Raman spectroscopy†

**DOI:** 10.1039/d2sc01967a

**Published:** 2022-08-30

**Authors:** Luca D'Amario, Maria Bruna Stella, Tomas Edvinsson, Maurizio Persico, Johannes Messinger, Holger Dau

**Affiliations:** Department of Chemistry, Ångström Laboratory, Uppsala University Box 523 751 20 Uppsala Sweden luca.damario@kemi.uu.se +46 18 471 6844 +46 18 471 6584; Department of Physics, Freie Universität Berlin Arnimallee 14 14195 Berlin Germany; Department of Chemistry and Industrial Chemistry, University of Pisa Via Moruzzi 13 56124 Pisa Italy; Department of Materials Science and Engineering, Uppsala University Box 35 751 03 Uppsala Sweden; Department of Chemistry, Chemical Biological Centre, Umeå University 90187 Umeå Sweden

## Abstract

Structural characterization of transient electrochemical species in the sub-millisecond time scale is the all-time wish of any electrochemist. Presently, common time resolution of structural spectro-electrochemical methods is about 0.1 seconds. Herein, a transient spectro-electrochemical Raman setup of easy implementation is described which allows sub-ms time resolution. The technique studies electrochemical processes by initiating the reaction with an electric potential (or current) pulse and analyses the product with a synchronized laser pulse of the modified Raman spectrometer. The approach was validated by studying a known redox driven isomerization of a Ru-based molecular switch grafted, as monolayer, on a SERS active Au microelectrode. Density-functional-theory calculations confirmed the spectral assignments to sub-ms transient species. This study paves the way to a new generation of time-resolved spectro-electrochemical techniques which will be of fundamental help in the development of next generation electrolizers, fuel cells and batteries.

## Abstract 2

Structural characterization of transient electrochemical species in the sub-millisecond time scale is the all-time wish of any electrochemist.

## Graphical abstract

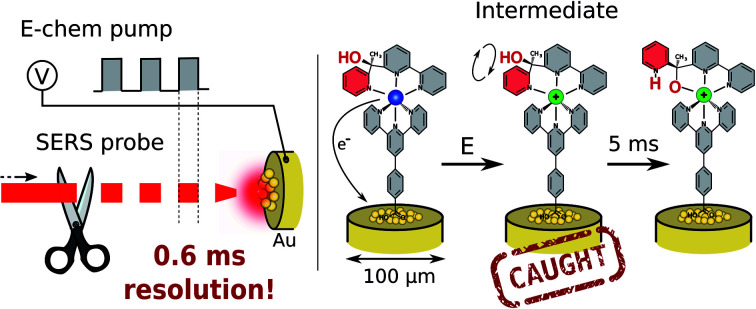

## 1 Introduction

One of the most fundamental goals of modern chemistry is to investigate reaction mechanisms down to their most elemental steps. In this regard, since its first application in the 50's, time resolved spectroscopy has been crucial in elucidating processes as diverse as vision, natural photosynthesis, electron transfer, DNA damage, heat transfer and quantum computing.^1–11^ In a typical time resolved spectroscopy measurement, the sample is excited with a pulse of light and the changes in its spectroscopic signal are monitored in time.^12^ To be able to perform such measurements the initial step of the studied process has to be photo-induceable.^13–15^ This is a limitation as the reaction under study might not be photochemical, or the sample not transparent. In addition, for chemical processes occurring as rare events, ultrafast transient spectroscopies have limited use, instead detailed spectroscopic information on species formed under micro- or millisecond time scales are preferred.

Electrochemical (e-chem) reactions are of great importance in modern technology, for example they are key to the functioning of batteries, electronic components, industrial production, electrolyzers and fuel cells.^16–19^ Despite their widespread use, the mechanisms of these reactions are difficult to study at fast time scales, *i.e.* by classical time resolved spectroscopy, because these reactions cannot or should not be photo-induced. Presently, chemico-physical information on e-chem processes are instead acquired, aside from electrochemistry, *via in situ* spectroscopic techniques, such as UV-vis spectroelectrochemistry, *in situ* Raman, IR and X-ray spectroscopy.^20–28^ In such methods an electrochemical cell is constructed such that a spectroscopic signal can be read from the surface of the electrode; the signal is measured while a given potential is applied. Usually, in these experiments, when the signal is recorded, the system is in a steady state; *e.g.* the measurement starts after a stable current reading is reached. In this way, no information on dynamics can be extracted. The signal originates from the average composition of the system at that given potential, *i.e.* from stable species. Thus, information on fast reacting species, in the ms time scale and faster, are lacking. This, generally speaking, hinders the understanding and development of e-chem processes and related devices; a classic example is the missing description of the dynamics of the lithium ion intercalation on Li-battery anodes.

Attempts to achieve time resolution with *in situ* techniques have been made.^29–32^ Commonly, a potential step is applied to trigger the e-chem reaction, while a series of spectra are recorded with short detector sampling time.^33–37^ With this strategy the *speed* of the detector is a crucial and limiting factor to time resolution. Moreover, limitations can also arise from a large time constant of the electrochemical cell.^37^ Among the techniques listed above, Raman spectroscopy is recently gaining popularity due to the simplicity of *in situ* implementation, its ability to give structural information and “high” time resolution. Common detectors, *e.g.* charge-coupled-device (CCD) cameras, have shortest sampling time of merely *ca.* 100 ms. This is a timescale of ion diffusion/migration, or macroscopic structural rearrangements like bubble formation. To be able to study reaction mechanism (bond breaking/formation) or ion exchange/intercalation or molecular structural rearrangements, time resolution of at least 0.1–0.01 ms is needed. Such a fast detection would require an upgrade to a purposely faster device, which however is expensive and not always possible. To the best of our knowledge, there is only one example of an *in situ* Raman setup (and *in situ* in general) that was equipped with a gated CCD camera that could reach 10–100 ms time resolution, by sampling the light only during short selected time periods.^35^ The downsides to this approach are, firstly, a low signal-to-noise ratio (S/N) necessitating extensive data collection, and secondly, the full potential of the camera (10–100 ns gating) cannot be utilized because the slow read-out limits its time resolution.

In this paper, simple (and unexpensive) modifications to a commercial confocal Raman spectrometer are reported that allow synchronisation of the Raman signal detection with the application of an electric pulse. The variation of the time period of this electric pulse allows to resolve transient intermediates, down to a maximum time resolution achieved presently of 0.6 ms. This is about 100 times faster than what was reached previously with an e-chem *in situ* structural spectroscopic method.^34,35^ As a test of the technique, a known electrochemical reaction is studied.

## 2 Results and discussion

### 2.1 Technique description

**Fig. 1:**
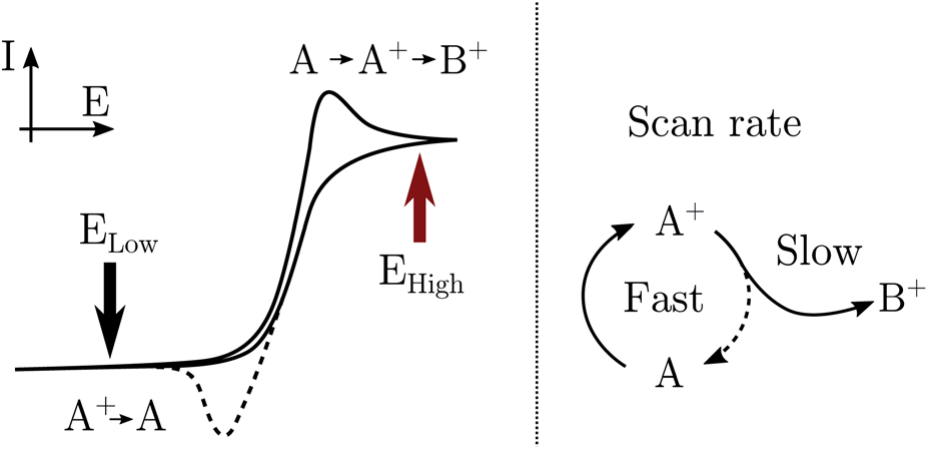
Schematic cyclic voltammogram of the A/A^+^/B^+^ system described in the text, in dashed lines the response obtained at scan-rates able to outcompete reaction A^+^ → B^+^. An “infinite pool” of the A species is assumed. On the right, the reaction scheme in function of scan-rates, fast *vs.* slow.

To be able to describe the working principles of the new technique presented here, the Electrochemical Induced Raman spectroscopy (EIR), it is useful to refer to a hypothetical electrochemical model. In the following, an irreversible EC system (electron transfer, followed by a chemical reaction) is considered:
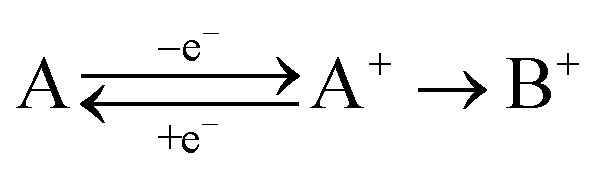
here a species A can be oxidized to the unstable species A^+^ which can convert to the stable species B^+^. A hypothetical CV of such system is reported in Fig. 1, showing a slow scan-rate experiment (solid line) and a fast one (added dashed line). Here “slow” and “fast” are taken in respect to the A^+^ → B^+^ reaction. It should be noted that, for clarity purposes, in Fig. 1 an “infinite pool” of the A species is assumed (*e.g.* reversible or diffusion controlled or catalytic systems), which assures the repeatability of the electrochemical cycle.

As shown in Fig. 1, in the slow scan, only an irreversible oxidation wave is present (with no reduction), while in the fast scan the potential sweep is fast enough to compete with the A^+^ → B^+^ conversion allowing the reduction of the A^+^ species. This system could be studied with classical *in situ* techniques, comparing spectra collected at two different potentials, *E*_low_ and *E*_high_, see Fig. 1. In such a hypothetical study, only the two stable species A and B^+^ would be observed, due to relative low concentration of the transient species in the steady-state conditions. In this example, the goal of the technique presented in this paper, would be to detect the unstable species A^+^.

The strategy adopted here is to apply a potential pulse of a given time period and amplitude to produce the wanted elusive state, say A^+^, and probe the system, *via* spectroscopy, only in this time period.

**Fig. 2:**
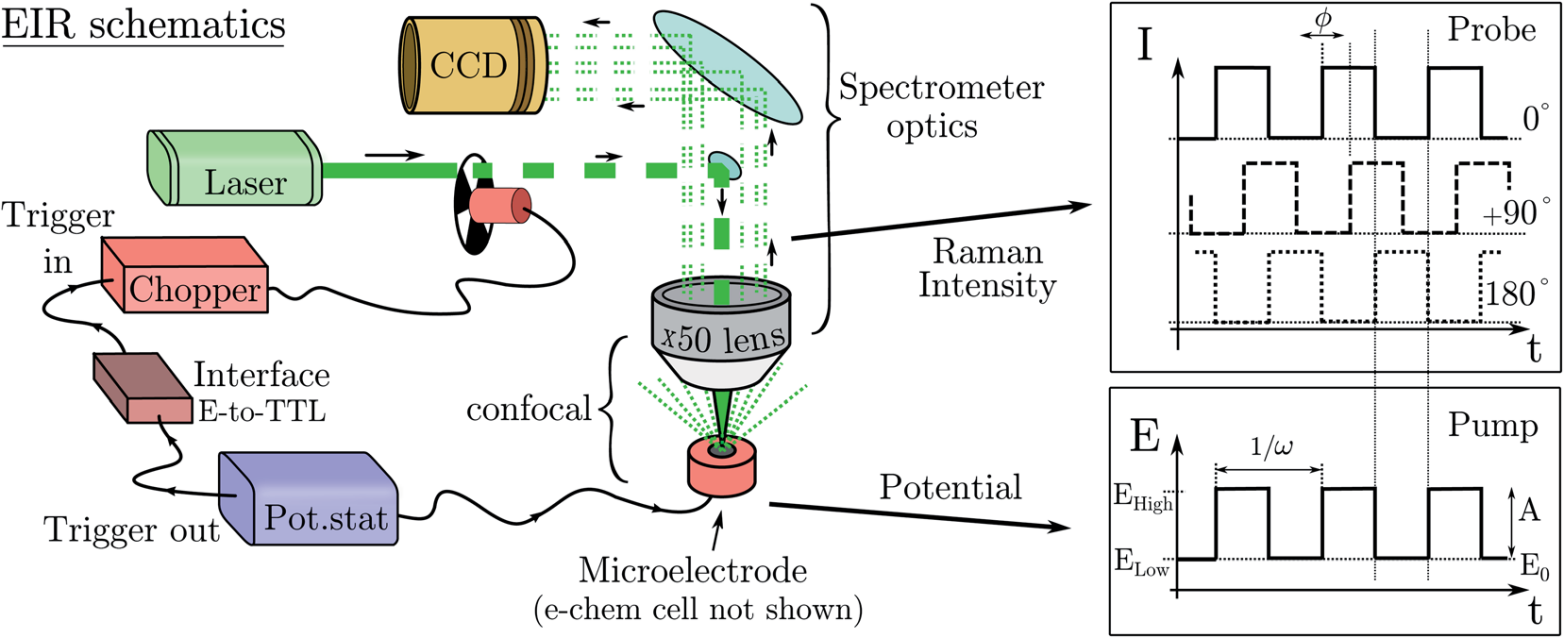
Scheme of the EIR setup with example of the modulation of Raman intensity and potential *vs.* time. Only the parts shown in RED are added to a usual Raman spectrometer to implement EIR. In the graphs, *ϕ* indicates the possibility to shift the phase of the Raman modulation (0°/90°/180°) with respect to the potential SW.

In practice, pumping is achieved by applying a train of potential pulses to an electrode (in an e-chem cell), *i.e.* a potential square wave (SW), see scheme in Fig. 2. The SW can be described by its frequency (*ω*), amplitude (*A*), phase (*ϕ*) and offset (*E*_0_) (potential bias). For practical reason, in the following discussion instead of amplitude and offset, the parameters “high” and “low” potentials will be used, respectively *E*_high_ and *E*_low_, see Fig. 2.

The electrode surface, where the reaction occurs, is placed in the focus of a Raman spectrometer (in microscope/confocal configuration) so that the Raman scattering coming from the surface of the electrode can be collected and analysed. The Raman laser intensity is also modulated at the same frequency of the potential modulation and is phase synchronised by a mechanical chopper, see Fig. 2. In this way the light that reaches the detector (CCD camera) is only the one that interacts with the sample in the desired pump SW state. Indeed, by changing the phase synchronisation, the Raman spectrometer can probe either the “high” potential (0° phase shift) or the “low” potential (180° phase shift) of the SW, see schemes in Fig. 2. The spectra of the high and low potential pulse state will be denoted H and L, respectively. The transient signal is given by the difference with a reference Raman spectrum (ref, which typically is the spectrum of the system without potential bias), *H*_r_ = *H* − ref. In the case of constant reaction rates during both the high and low crests of the SW, the 0/180° delta Raman, might not be informative due to similar average compositions. In these cases spectra recorded at ±90°, which probes the rising (*R*, −90°) or the falling (*F*, +90°) edge of the SW, are the most descriptive cases, see Fig. 2 and S3 in ESI.†

The periodic nature of the pump requires that the system in study should be able to restore, at least partially, to its initial condition at every period.

**Fig. 3:**
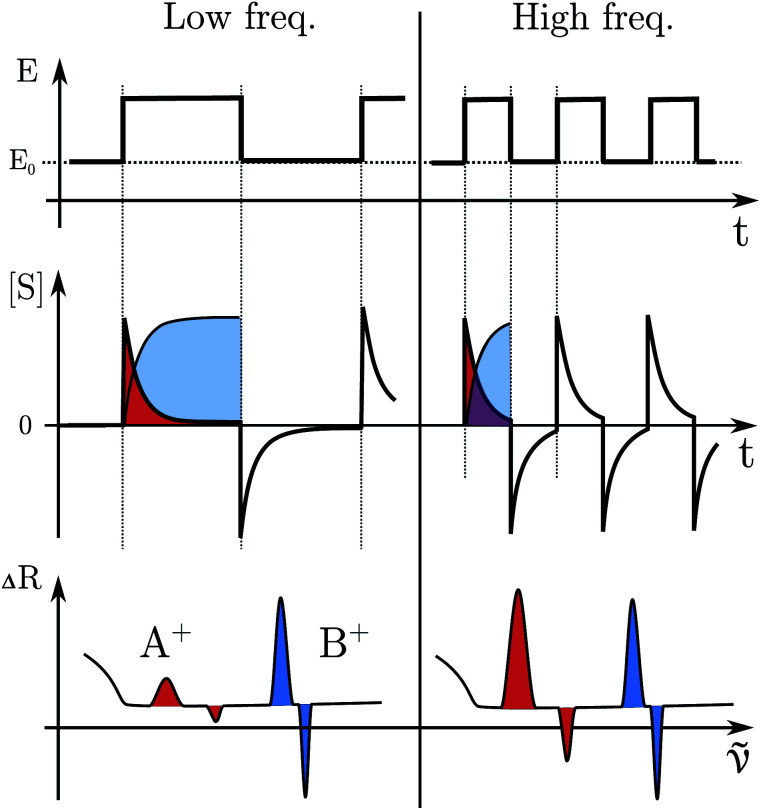
Expected data from an EIR measurement; comparison between low and high frequency results. In the top, the pump SW; in the middle, the evolution of the species concentration; in the bottom, the Raman difference spectra. Areas in red represent the signal from a fast evolving state, those in blue a slowly evolving or stable one.

The formation or breaking of a bond can be visible in the Raman signal which can provide structural information of the species that are evolving between the high and low state of the SW, see Fig. 3. In particular, fast evolving phenomena will be more visible in the spectra recorded at high SW frequencies (short pump/probe pulses) in respect to the slow phenomena that will be visible at any frequency, see Fig. 3.

#### EIR technical implementation

In the configuration that is described here, EIR reaches the time resolution of about 0.6 ms (SW frequency of 834 Hz). In the following, the main technical implementation details are given, for further detail see the ESI.†

#### Electrode assembly

**Fig. 4:**
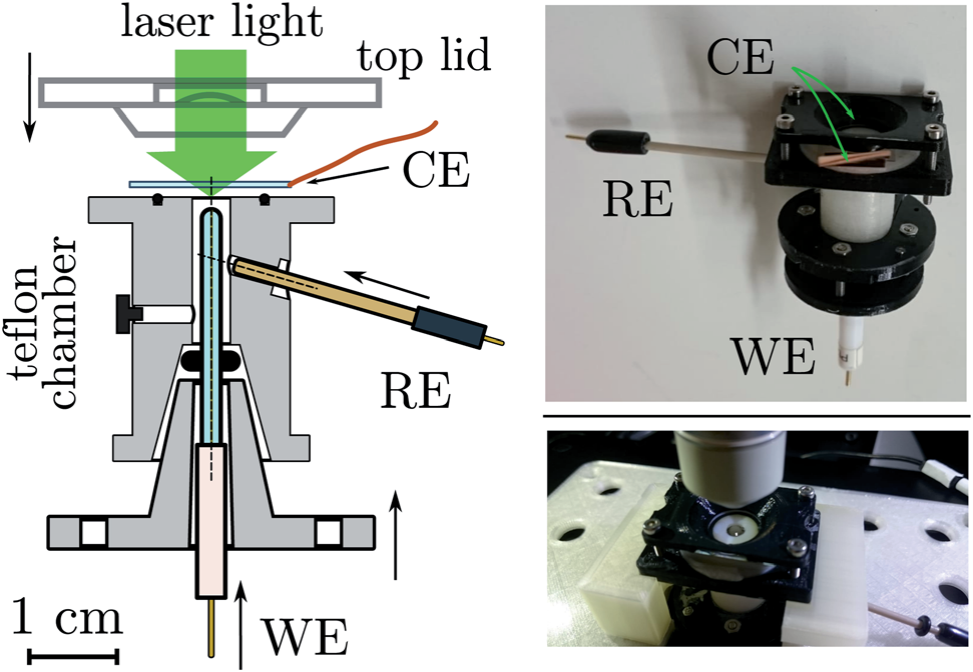
(Left) Section of the e-chem cell design (Teflon made), see Fig. S1 in ESI,† for detailed technical sketches. The WE and RE electrodes are shown in the final positions, for clarity the CE is not attached to the cell. Supporting flanges for closure are not shown. (Right) Pictures of the real cell.

Time accuracy of the potential application at the electrode is limited by the time constant of the electrochemical cell *τ* = RC where R is the uncompensated resistance and C is the electrode capacitance. By using microelectrodes, which feature a flat active area of *ca.* 25–100 μm in diameter, RC can be largely reduced (to 10^−7^ s). The use of microelectrodes is particularly suited in this case since the Raman beam in microscope spectrometers can easily be focused in such a small area (10–50 μm). This ensures that the information given from the electrochemical and the Raman measurements regard the same portion of the sample. Gold and platinum microelectrodes (50 or 100 μm diameter) were built in-house as described in the experimental section. The electrode was mounted in the customized e-chem cell schematized in Fig. 4 and S1,† in a vertical configuration with the electrode surface facing the spectrometer objective, see photo in Fig. 4. The counter electrode (CE), a platinized 0.1 mm thick fluorine-doped-tin-oxide (FTO) transparent sheet (16 × 20 mm^2^), functioned also as entrance window for the probe laser light. The electrolyte volume was about 50–150 μL.

#### Raman beam modulation

The laser modulation needs to have exactly the same frequency of the pump and be synchronised in phase. Commercial Raman spectrometers do not offer the possibility to modulate the laser beam (CW lasers), hence the modulation has to be implemented by additional hardware.

A light chopper can be easily placed in the laser path in any position before the beam enters the spectrometer and also be used for any of the lasers installed in the Raman set-up. The chopper controller can be synchronised (in frequency and phase) with the potentiostat, see Fig. 2. The Raman signal probes similar time scales of the width of the potential pulse, thus the time resolution is limited to the shortest period of the SW/Raman modulation. In this regard, the possibility to use instead (ns) pulsed lasers was discarded since many of these systems can mode lock differently in between the pulses, which causes several nanometers shift of the laser emission band; this, disqualifies them for use as a Raman laser which requires a highly monchromatic and stable laser line.

#### Signal detection

The collection of the signal is carried out by the same detector as used in steady-state Raman spectroscopy. The Raman scattering is normally detected by a CCD camera that integrates the signal over several seconds. In the here described EIR method, the CCD receives and integrates the modulated signal from the chopped light over seconds, as usual. The read-out times of the CCD camera do not affect the time resolution since this is given by the probe beam modulation. The time resolution is enabled by the probe pulse and not by the speed of the detector, in analogy to the design principles in ultrafast spectroscopy. Moreover, since the Raman pulses illuminate the detector with the half of the light flux of a continuous-light Raman experiment, the signal/noise and the collection time in EIR is similar to those of a steady-state Raman measurement, which is a great advantage of this technique. In summary, time resolution can be achieved without modification of the standard detection system of the Raman spectrometer, at favorable signal-to-noise ratio.

#### Pump–probe synchronisation

The potentiostat was synchronized with the chopper controller *via* a programmed microcontroller (MyRIO), see Fig. 2 and ESI† for further details. The response in time of the MyRIO is crucial for controlling the phase between the pump SW and the probe laser light. A successful test of the phase control was conducted measuring the output of the chopped laser intensity *vs.* the output of the reading of the electrode SW potential (the real applied potential), see Fig. S2 in ESI.†

### 2.2 Validation

**Fig. 5:**
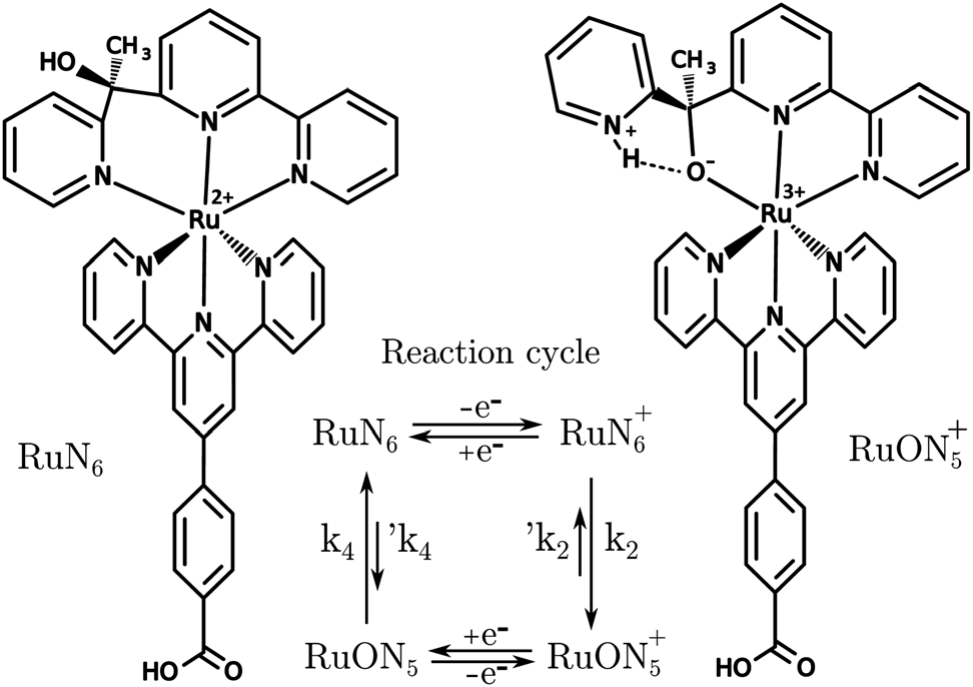
Structures of RuN_6_, [Ru(HOOC–Ph-tpy)(1-[6-(2,2′-bipyridyl)]-1-(2-pyridyl)ethanol)]^2+^, and RuON_5_^+^ with related reaction cycle of the electron transfer induced isomerization. *k*_2_ and *k*_4_ are *ca.* 150–200 and 500–1000 s^−1^, respectively (e^−^ transfers occur in *ca.* 10^4^ s).^39^

A known Ru based molecular switch, RuN_6_, see Fig. 5, was selected to test the EIR technique.^38–40^ This system was chosen due to its rare combination of characteristics: it is reversible and highly stable, with a known reaction mechanism and desired kinetics (*ca.* 200 s^−1^).

**Fig. 6:**
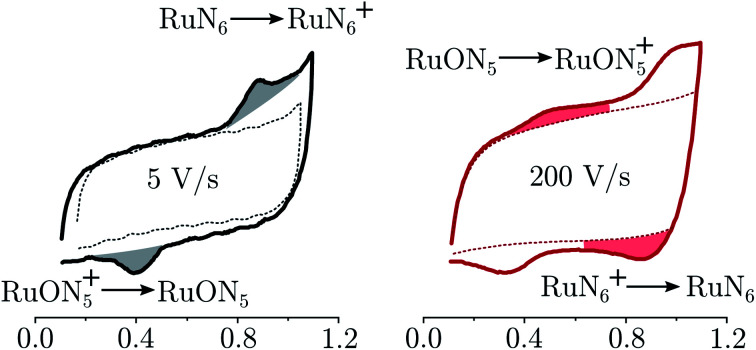
Electrochemistry of RuN_6_ grafted on Pt microelectrode, at two scan-rates 5 and 200 V s^−1^. In dashed lines the blank electrochemistry (without complex) to account for the coulombic current. The grey areas indicate the irreversible waves due to the stable species; the red areas indicates the redox waves due to the unstable species, visible only at high scan-rates.

RuN_6_ is a hexapyridyl coordinated Ru(ii) complex that shows structural bistability depending on the Ru center oxidation state. The switching ligand, here abbreviated “BpyPyEt”, favores an N bond to Ru(ii) while it binds *via* O to Ru(iii); the full isomerization cycle is shown in Fig. 5. The isomerization can be followed by microelectrode cyclic voltammetry at different scan rates, see Fig. 6 and S4.† At low scan rates the CV shows an irreversible redox behaviour originating from the two stable species RuN_6_ and RuON_5_^+^; while at fast scan rates, where the reverse redox reaction competes efficiently with the isomerization, the CV shows two reversible redox couples, *i.e.* all the four species (see ESI† for a detailed treatment supported by e-chem data).

Lomoth *et al.* have shown that the isomerization kinetics can be tuned by changing the alkyl substituent of the bridging carbon of the pyridil-C-bipyridil ligand.^39^ With the methyl substituent used here, the RuN_6_ shows a kinetics of about 200 s^−1^ which is on the timescale reachable by the EIR, but hardly by any other spectroelectrochemical technique. Indeed, the isomerization mechanism has been proven only *via* electrochemical methods supported by computational simulations. We emphasize that in the EIR experiment, not only does the basic Raman-scattering detection scheme provide high time resolution, but also the spatial resolution is on the order of few micrometers, allowing the use of microelectrodes that support sub-microsecond electrochemical response times. Here we can directly probe the isomerization by Raman spectroscopy, thereby illustrating the potential of the new EIR methodology. Namely, at low pump SW frequencies, only the stable RuN_6_ and RuON_5_^+^ spectra should be visible in the spectra, while at high frequencies the transient species RuN_6_^+^ and RuON_5_, should also appear.

RuN_6_ was loaded on a (*Φ* = 100 μm) gold electrode functionalized with gold nanoparticles (AuNP) to enable surface-enhanced Raman spectroscopy (SERS). Linkage occurred through amino-thiol chains, as the following reaction sequence summarizes:
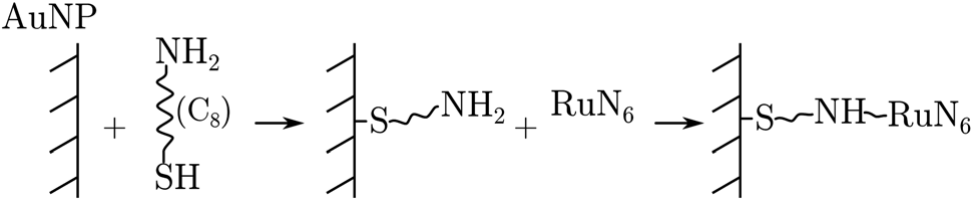


This results in a monolayer coverage of the electrode area. A purposely designed Raman imaging measurement of the functionalised electrode was carried out to localize enhancement hotspots (see ESI†); which revealed that the majority of the hotspots are located at the edge of the microelectrode. EIR spectra were then collected using a slightly defocused beam spot (to increase its size) placed at the edge of the electrode, see the ESI† for further details.

#### EIR spectra prediction by DFT

**Fig. 7:**
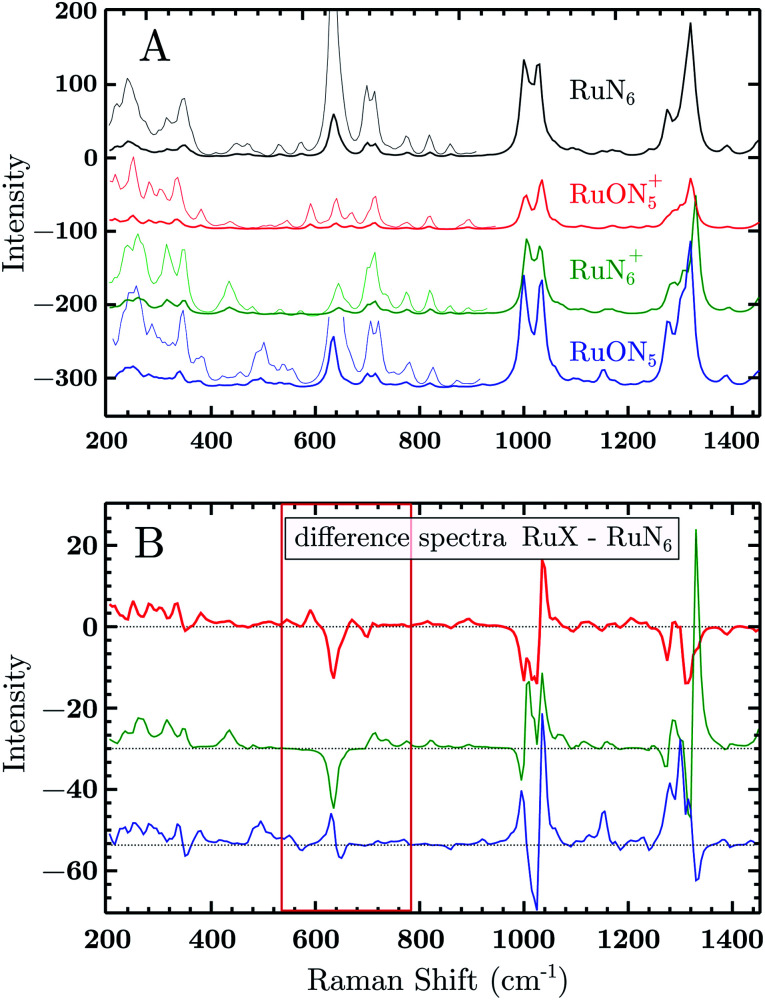
(A) Vibrational Raman spectra of the RuN_6_ family calculated by DFT. The spectra were produced by a sum of Lorentzian functions (15 cm^−1^ FWHM) constructed from the frequencies and amplitudes of the simulated modes. The intensities in the region 150–800 cm^−1^ were magnified ×5 for clarity. (B) Difference spectra calculated from above data subtracting the RuN_6_ spectrum from the other three. The spectral range of main interest has been highlighted. The spectra are offset for clarity.

Due to the complex structure of the model system, in order to facilitate data interpretation, the Raman spectra of the four species were simulated by density-functional-theory (DFT) calculations. The resulting mode frequencies and intensities are plotted in Fig. 7A (further details are discussed in the ESI†). A general rough assignment of the most prominent modes can be done as follows. The bands at low frequencies, 200–400 cm^−1^, are assigned to whole rings displacements (respect to Ru). The bands at 600 and 750 cm^−1^ are assigned to ring stretching modes (comprehending the Ru–N stretching bonds) of the BpyPyEt and terpyridine ligands in the RuN_6_ isomer. At around 1000 cm^−1^ and 1300–1600 cm^−1^ one can find the C–H in-plane and out-of-plane bending and ring C–C stretching, respectively. By examining the simulated spectra, it can be seen that the frequency and intensity of some of these modes are affected by Ru–O/N bond isomerization and/or from the Ru oxidation state. One clear example are the modes at 600–750 cm^−1^, being directly correlated to the Ru–N stretching modes. Other modes, even though not directly related to a Ru–O/N displacement, are affected by the isomerization due to a change in geometry/symmetry of the molecule, like the modes around 1000 cm^−1^ (C–H bend) and 1300 cm^−1^ (C–C stretching), see Fig. 7A.

To test the accuracy of these calculations we compared the RuN_6_ DFT spectrum with the experimental powder spectrum (Fig. S10†). These agree within ±30 cm^−1^, which is sufficient for our scope. We additionally analyzed the effect of H/D substitution (Fig. S10†), which confirms that relevant peaks, for species discrimination, can be found at 600–750 cm^−1^, 1000 cm^−1^ and around 1300 cm^−1^.

Based on electrochemical kinetics analysis and taking RuN_6_ as reference (the stable species at open circuit potential, ocp), a rough estimation of the experimental difference spectra can be performed. Considering that the maximum EIR pump semiperiod is 0.6 ms and that the isomerization constants are 150–200 s^−1^ and 500–1000 s^−1^, for *k*_2_ and *k*_4_ respectively, it can be predicted that the transient RuN_6_^+^ species (green spectrum in Fig. 7A) should be observed, while the RuON_5_ isomer (blue spectrum) might not be. In order to emulate the EIR difference spectra, the calculated RuN_6_ spectrum has been subtracted from the spectra of the other three species to produce the difference spectra in Fig. 7B. Following the predictions just listed, the difference spectra at mid-low pump frequency should resemble the RuON_5_^+^–RuN_6_ (red) spectrum in Fig. 7B (*e.g.* with mainly negative features around 650, 1050 and 1300 cm^−1^); while at fast pump frequencies, new difference (positive) features should appear at around 710, 1000, 1300 cm^−1^, resembling the RuN_6_^+^–RuN_6_ (green) spectrum in Fig. 7B.

**Fig. 8:**
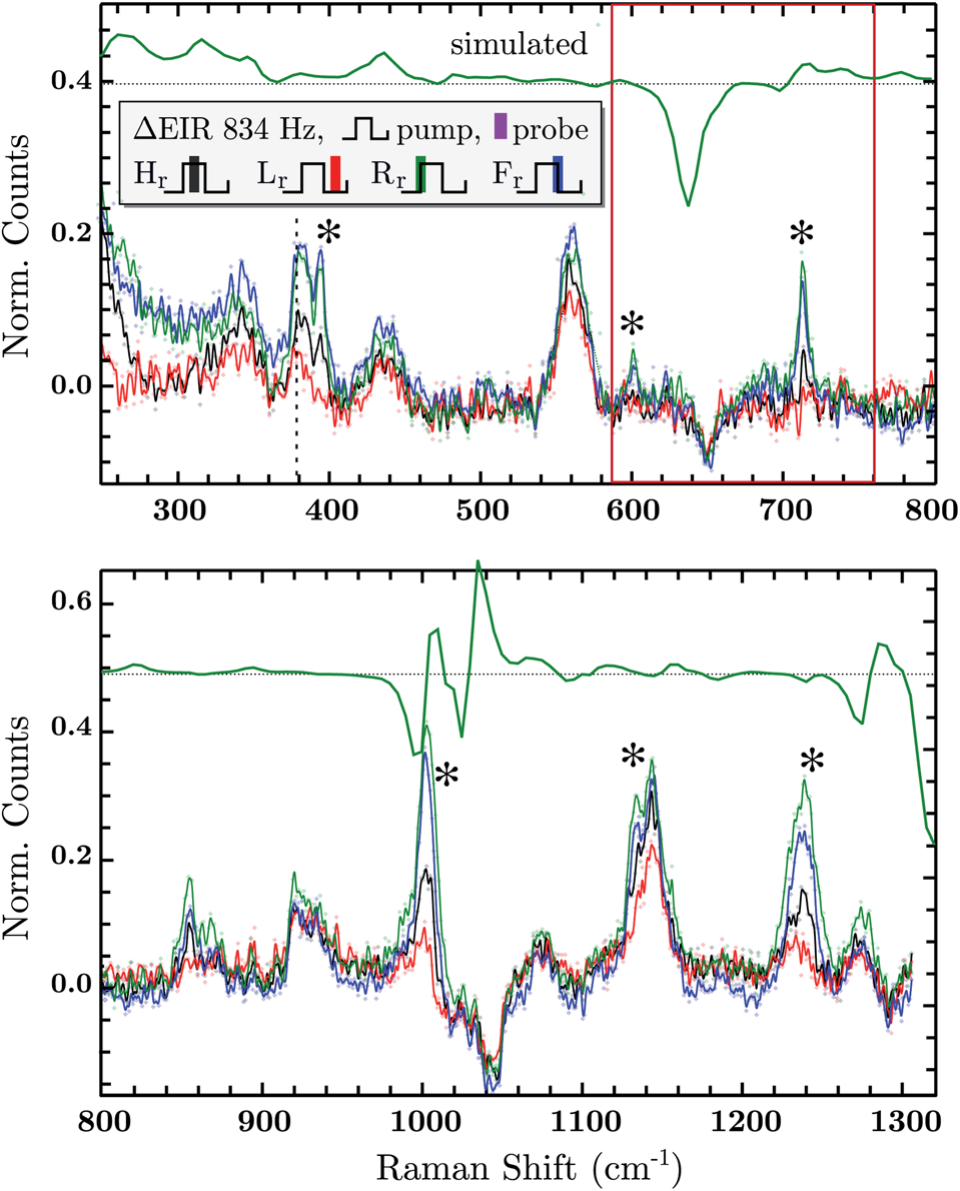
Difference EIR spectra of the RuN_6_ functionalised AuNP electrode of the 834 Hz measurement in all four phase shifts, the ocp spectrum has been taken as reference (subtracted and normalized). EIR phases: *H*_r_ black, *L*_r_ red, *R*_r_ green, *F*_r_ blue. The *R*_r_ trace from numerical simulation is reported in green, shifted for clarity. Discussed spectral range has been highlighted for clarity.

The DFT calculated spectra have been used to perform a numerical simulation of the EIR difference spectra in order to accurately predict the experimental results. The simulation was carried out operating a weighted integration of the kinetics laws of all the evolving species to calculate every contribution in each EIR spectrum. The weighting law resembles the shape of the probe pulse train. The full simulation is reported in the ESI.† Here only the one relevant spectrum is shown, the 834 − *R*_r_ difference spectrum (which is similar to the *H*_r_ and *R*_r_, see Fig. S21†), reported in comparison to the experimental data, in Fig. 8. It can be seen that the 834-*R*_r_ simulated spectrum, *i.e.* the *R*_r_ spectrum at the highest time resolution, indeed resembles the RuN_6_^+^–RuN_6_ green spectrum in Fig. 7B, as intuitively predicted above.

#### EIR measurements

Experimental EIR spectra of the RuN_6_ grafted electrode, were collected using a 785 nm laser and setting the pump SW frequency to 20 Hz, 200 Hz and 834 Hz (semiperiods of 25, 2.5 and 0.6 ms respectively), using 0 V_ref_ as *E*_low_ and 0.9 V_ref_ as *E*_high_, see Fig. S16.†

Delta EIR spectra have been calculated, by treating the raw spectra according to literature protocols, to account for a different magnitude of enhancement.^41^ Briefly, the flat background *B* is subtracted from the raw spectra *R* and is also used to normalize them, the final spectra being *S* = (*R* − *B*)/*B*. The spectrum recorded at ocp (reported in Fig. S16†) has been taken as reference which was subtracted from the 20 − *H* (spectrum recorder at 20 Hz pump frequency, “high” phase) and 834 − *H* spectra producing the results reported in the ESI, Fig. S17.† Here we report only the most significant difference spectra, *i.e.* all phases *H*_r_/*L*_r_/*R*_r_/*F*_r_, recorded at the fastest pumping frequency, 834 Hz, in Fig. 8.

In Fig. 8, negative difference bands can be found: at 650 cm^−1^; a broad band at 1050 cm^−1^; and a final at *ca.* 1300 cm^−1^. These negative features derive from the subtraction with the ocp spectrum that was taken as reference, see Fig. S16.† Relevant positive difference bands (indicated with asterisks) are found at: 392; 600; 712; 1000; 1140 and 1230 cm^−1^. Other positive bands are of less interest since they are present in all EIR difference spectra of all pumping frequencies, again derived from differences with the ocp spectrum. These omnipresent positive and negative bands are likely due to slowly evolving transient species (*i.e.* species that appear/disappear only on a fully rested system, at ocp). This is supported by the electrochemical kinetic measurements, which show a small, slow (6.7 s^−1^) component in the isomerization kinetics, see ESI† for further details.

As predicted, the 834 − *H*_r_ spectrum resembles the 20 − *H*_r_ spectrum, see Fig. S17,† meaning it does not display clear transient features. Transient bands can be seen in the ±90° (*R*_r_ and *F*_r_, green and blue respectively), that are not present or are not as strong in the 0/180° (*H*_r_ and *L*_r_, black and red). This is expected since, as mentioned earlier, depending on conditions, the ±90° phase spectra can probe faster evolving processes, *vide infra*.

The experimental delta spectra, in Fig. 8, are expected to be a combination of all the four species. This is due in part to the kinetics of the system and in part to the not perfectly squared shape of the probe pulses. The pulses show a rising/falling components that mix the H and L signals around the edges, see ESI† for a thorough clarification regarding this topic. Indeed, it can be seen that each of the EIR spectra contains features of the calculated spectra in Fig. 7. However, it is also clear that the new features that arise in the ±90° spectra resemble mainly the RuN_6_^+^–RuN_6_ (green) difference spectrum in Fig. 7B, as well as the simulated 834 − *R*_r_ spectrum in Fig. 8, with remarkable accuracy in the 250–800 cm^−1^ region. In Fig. 8, a sharp positive feature, at 712 cm^−1^, absent in the *L*_r_ difference spectrum, is present with medium intensity in the *H*_r_ spectrum and quite prominent in the *R*_r_ and *R*_r_ difference spectra. In the same region, a broad negative band at around 650 cm^−1^ and a small positive peak at 600 cm^−1^ can be observed. Altogether these three peaks can be constructed by the sum of the computed different spectra, red, green and blue in Fig. 7, with prevalence of the green spectrum. Overall this leads to assign the 712 cm^−1^ peak to transient species. Even though the 712 cm^−1^ peak can be attributed to both unstable isomers, RuN_6_^+^ and RuON_5_ (this last one with weaker amplitude, see Fig. S19†), they are both transient intermediates visible only at sub-ms timescales, proving the effectiveness of the EIR technique.

Other parts of the spectrum, *e.g.* below 400 cm^−1^ or around 1000 or 1300 cm^−1^ are more fluctuating or similar among the species to be considered here, they are discussed in detail in the ESI.†

**Fig. 9:**
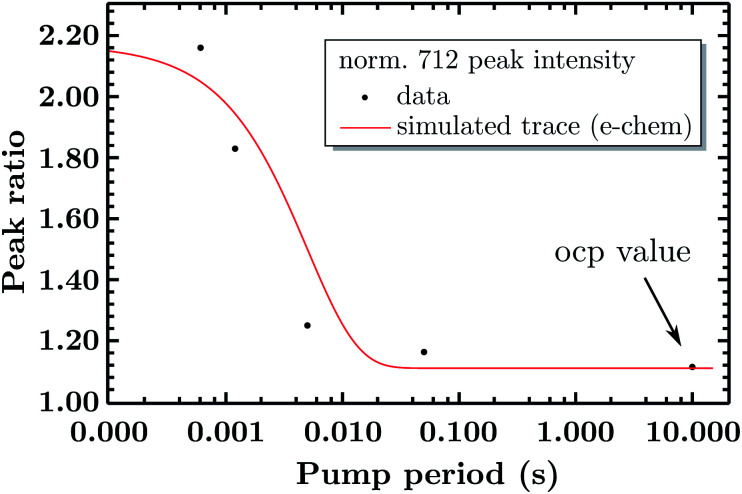
Comparison of the experimental amplitude ratio of the normalized 712 peaks over time and its simulated trace (as a first order reaction) of the measured kinetics (200 s^−1^).

So far the spectra have been analyzed regarding informative spectral features, though the EIR technique can also give kinetic information (rate constants) by peak magnitude *vs.* pump frequency analysis. A mathematical model able to deconvolute EIR spectra on the basis of kinetic laws is under development. Here, a simple comparison of single spectra with the predicted kinetics can be performed. The peak at 712 cm^−1^ has been taken as example for comparison with known kinetic data. The normalized amplitude (*vs.* the 670–690 cm^−1^ band, raw spectra) of the 712 cm^−1^ peak is plotted in Fig. 9 as a function of pump semiperiod, together with the expected kinetic trace from a first order reaction with 200 s^−1^ rate constant. The results confirm that the experimental findings can be described by the reaction-kinetic model described in Fig. 9.

#### EIR versatility

EIR is a versatile technique that can be applied to study a large range of e-chem systems. The e-chem cell is the only part to modify in order to adapt the EIR method to other systems. In this regard, a few factors should be taken in consideration:

• The electrode area should be small to ensure fast pump potential response.

• Any electrode material can be used provided having enough scattering from the sample. SERS inactive substrates have been tested and promising preliminary results were obtained with solid inorganic layers (like Co oxides for Li-batteries).

• For reactions involving gas evolution (*e.g.* water oxidation) a flow cell is needed to remove bubbles from the electrode.

• Spectral simulation is not strictly necessary to interpret EIR results if experimental reference spectra are available or the EIR spectra are simple enough to be interpreted *via* isotope substitution or fundamental frequency estimation.

## 3 Conclusion

This report describes and demonstrates the validity of EIR technique, which is the first to combine structural spectroscopy with a pulsed electrochemical protocol achieving sub millisecond time resolution. This enables the effective detection of electrochemical reaction intermediates. We expect this new technique to have a large impact, because of its straight forward adaptation to a large number of important research fields, including batteries and electrocatalysis for renewable energy and circular economy.

## 4 Experimental details

Fast electrochemistry was performed with an Autolab PGSTAT302N equipped with a Scangen and DAC750 modules for fast analog-to-digital conversion. Working microelectrodes for fast e-chem were prepared in-house from Pt or Au wire (50 or 100 μm diameter) and borosilicate glass capillary. Alumina, 0.3 and 0.05 μm diameter, was used to fine polish the metal surface. A graphite rod was used as counter electrode and a Ag wire in a 10 mM AgNO_3_ solution as reference electrode.

Au nanoparticles were synthesized by negatively polarizing a clean surface Au microelectrode, with −0.17 V, in a solution of HAuCl_4_ 0.1 M, in a two electrode cell, for a period of time (typically 5 s), repeated 5 times. Such microelectrode was electrochemically polished by applying, in a two electrode cell, ten cycles between −0.4 and 1.4 V in a 0.5 M H_2_SO_4_ solution. The electrode was rinsed with DI water (>18 MΩ cm^−1^), immersed in a 5 mM solution of 8-amino-octanthiol (8AT) for 30 min, the 8AT functionalized electrode was rinsed and immersed in a solution of the Ru-complex, 5 mM, and 10 mM 4-(4,6-dimethoxy-1,3,5-triazin-2-yl)-4-methylmorpholinium chloride (DTDMM) catalyst for *ca.* 48 h.

Raman spectroscopy was performed with an inVia spectrometer by Renishaw equipped with three lasers, 473 nm, 532 nm, 785 nm. The 50× objective from Leica was used in the recording of dry samples while a 63× water immersion objective from Leica was used in the EIR and *in situ* measurements. The phase locked chopper was purchased from Thorlabs (MC2000B); the TTL interface was programmed *via* a NI MyRIO-1900. Further details are reported in the text and ESI.†

Raman spectra simulations were performed on the optimized molecular geometries. Density Functional Theory (DFT) was employed for the electronic calculations, using the B3LYP exchange–correlation functional.^42^ The ‘Los Alamos National Labs' effective core potentials were used for the Ru atom.^43^ The basis set was of double-ζ type (LANL2DZ)^43,44^ with the addition of polarization functions on the heavy atoms.^43,45,46^ Solvation effects were included by means of the polarizable continuum model (PCM). For more details, see ESI.†

Details on used materials and compounds are reported in the ESI.†

## Data availability

All relevant data is presented either in the article or as in the ESI.† The original data in form of text files can be provided by the corresponding author upon reasonable request.

## Author contributions

The EIR technique was conceived, designed and implemented by LD. Experiments were performed and analyzed by LD with support of MBS and TE. DFT calculations were performed by MBS and MP. The manuscript was written by LD, JM and HD, with contributions from TE, MBS and MP.

## Conflicts of interest

The authors declare no coflict of interest.

## Supplementary Material

SC-013-D2SC01967A-s001
