# Supplementary material for: Towards time resolved characterization of electrochemical reactions: electrochemically-induced Raman spectroscopy
Source: Chem Sci. 2022 Aug 30;13(36):10734–42. doi: 10.1039/d2sc01967a (PMC9491093; doi:10.1039/d2sc01967a)
Supplement: SC-013-D2SC01967A-s001 [file SC-013-D2SC01967A-s001.pdf]

Electronic Supplementary Material (ESI) for Chemical Science.
This journal is © The Royal Society of Chemistry 2022

Supporting Information: Towards time resolved characterization of electrochemical reactions: Electrochemically-Induced Raman Spectroscopy.

Luca D'Amario,[*,†,‡] Maria Bruna Stella,[¶] Tomas Edvinsson,[§] Maurizio Persico,[¶] Johannes Messinger,[†,k] and Holger Dau[‡]

†*Department of Chemistry - Ångström Laboratory, Uppsala University, Box 523, 751 20 Uppsala, Sweden*

‡*Department of Physics, Freie Universität Berlin, Arnimallee 14, 14195 Berlin, Germany*

¶*Department of Chemistry and Industrial Chemistry, University of Pisa, Via Moruzzi 13, 56124 Pisa, Italy*

§*Department of Materials Science and Engineering, Uppsala University, Box 35, 751 03 Uppsala, Sweden*

k*Department of Chemistry, Chemical Biological Centre, Umeå University, 90187 Umeå, Sweden*

E-mail: luca.damario@kemi.uu.se

Phone: +46 737217100. Fax: +46 (0)18 4716584

Contents

Custom e-chem cell design S-3

Potentiostat-Chopper synchronisation. S-3

Potentiostat-chopper synchronisation test S-4

MyRIO algorithm and benchmark S-5

180° vs. 90° phase shift. S-6

RuN_6 electrochemistry S-7

Powder Raman spectra S-10

Raman imaging to locate/quantify hotspots. S-11

Computational spectra S-14

Control spectra S-15

H/D isotope substitution. S-15

Amino-thiol linkers spectrum. S-16

Raw EIR spectra S-17

Complex in solution. S-17

Complex grafted on electrode. S-18

Detailed discussion of spectra. S-21

Kinetic trace and EIR spectra simulations S-26

Further material details S-31

Reading note: figures that refer to the main paper are indicated as "Fig. n" while the ones that refer to the Supporting Information are named "Fig. Sn".

Custom e-chem cell design

A cylindrical teflon piece worked as a container surrounding the working microelectrode (WE), a leakless Ag/AgCl reference electrode (RE) was loaded from a side aperture allowing 3 mm distance from the WE to minimise uncompensated resistance. The counter electrode (CE) functioned as entrance window for the probe laser light, see scheme in Fig. 4 in main paper, thus it was constructed with a platinized 0.1 mm thick fluorine-doped-tin-oxide (FTO) transparent sheet (16x20 mm^2). Platinization of the FTO was conducted as described elsewhere.[?] The WE-CE distance was about 2 mm to be able to use a 3 mm working distance objective (63x, water immersion objective). All the entrances were kept sealed via o-rings. The electrolyte volume was about 50-150 μL (depending on the thickness of the WE supporting glass). Here the technical sketches of the main chamber, which were omitted in the main description for clarity purposes, are reported in Fig. S1.

Potentiostat-Chopper synchronisation.

The potentiostat used in this work as EIR pump, has a useful AUX port that allows to read in real-time the applied potential (few ns error in time). This is quite convenient to implement the synchronisation with the chopper controller. The chopper controller receives TTL pulses as reference for the frequency and phase. Since the pump potential can be any couple of E_{High} and E_{Low} the AUX output from the potentiostat has to be amplified to be used as a TTL reference, see Fig 2 in main paper. To facilitate the usability of the technique a programmable DAC device was used (NI MyRIO), that continuously read the output of the potentiostat and converted it into a TTL signal by comparison with a user-set threshold (usually set as $(E_{High}-E_{Low})/2$). The maximum sampling of the MyRIO is 500 kS/s. Assuming that 10 points per cycle are needed to preserve time precision, the maximum EIR frequency reachable with this configuration is 50 kHz. Though, the mechanical chopper used here can reach 10 kHz, which fixes

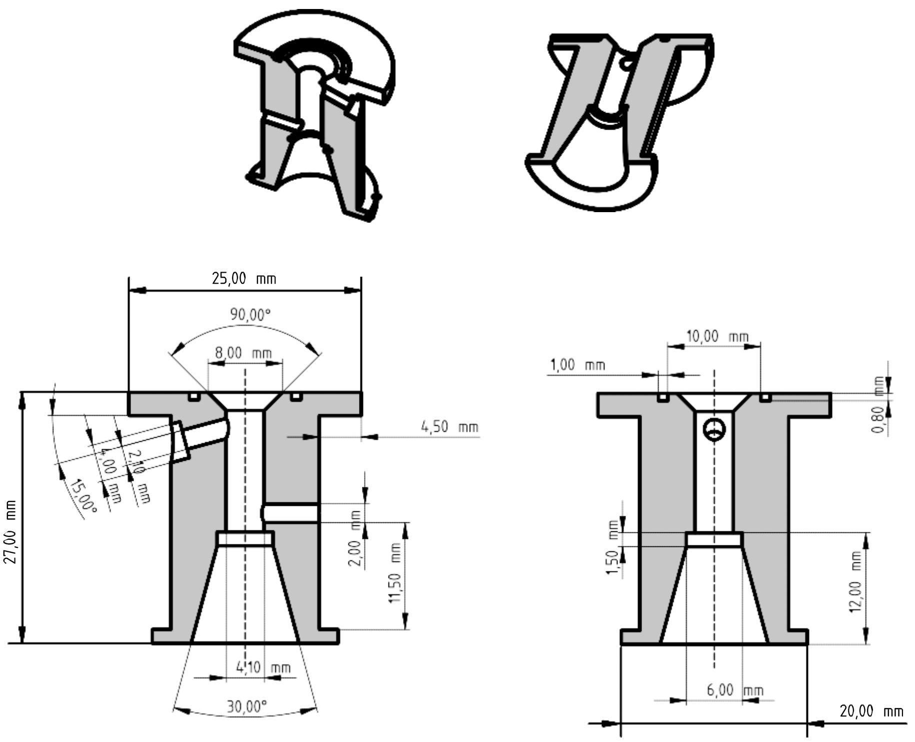

Figure S1: Technical sketches of the echem. On the top two half-sections of the solid piece are given. In the bottom the details of the piece dimensions are reported.

the current maximum time resolution (0.1 ms). Implementation of a faster probe laser modulation is presently ongoing in our lab.

The response of the MyRIO interface is crucial to the control of the phase between the pump SW and the probe laser light. A delay of the MyRIO on the E-to-TTL conversion would produce an error in the pump/probe phase. Tests of the phase control were successfully conducted, see next section.

Potentiostat-chopper synchronisation test

The synchronisation between the potentiostat pump square wave (SW) and the actual laser light modulation was tested for time resolution benchmark. To this scope a fast Si detector (DET10 from Thorlabs, 10 ns rise time) was placed in the laser path after light modulation as shown in Fig. S2. An oscilloscope read the signal coming from the detector triggered by the reference signal taken from the potentiostat (that produced the

pump SW). The e results of the test (the oscilloscope reading) are reported in Fig. S2. A time window of 2 ms shows that the phase is constant during such time period, a longer test (not shown) showed that the phase can be kept constant indefinitely (over hours time scale), confirming the specification of the phase locked chopper. The magnification of the oscilloscope reading show the shape of the light pulse in comparison to the pump potential SW. The feedback loop of the chopper controller induces a unavoidable error in the phase while performing the continuous phase synchronization. This can be seen in the slight mismatch of the signals phases over the 2 ms signal. This was estimated to bring a <3% error in the EIR spectra. This test was repeated also with a different chopper wheel that allows the chopper to reach 10 kHz chopping frequency. The test was successful but results are not reported here since it was considered not relevent due to the different modulation profile induced by the different wheel. The faster wheel makes the modulation sinusoidal (opposite to squared), with a minimum (zero light) centered in the middle of the low potential and the maximum (100% light) in the middle of the high potential. This is currently being evaluated whether is useful for EIR purposes, thus was not included in the main paper. If accepted this would bring the time resolution of EIR up to 50 μs.

MyRIO algorithm and benchmark

The potentiostat-chopper interface, the NI MyRIO, was programmed with a very simple algorithm to assure the fastest response. The algorithm can be summarized as: read potentiostat potential; if it is lower than a (given) ***threshold*** apply 0 V to the output; if it is higher than ***threshold*** apply 3.3 V. MyRIO has a analog-to-digital (DAC) conversion rate of 500 kS/s with a processor clock of 667 MHz, which ensures the signal-to-TTL conversion occurs in about 2 μs. This was tested and confirmed, which ensure that the current time resolution limit of the EIR is not given by the potentiostat-chopper interface.

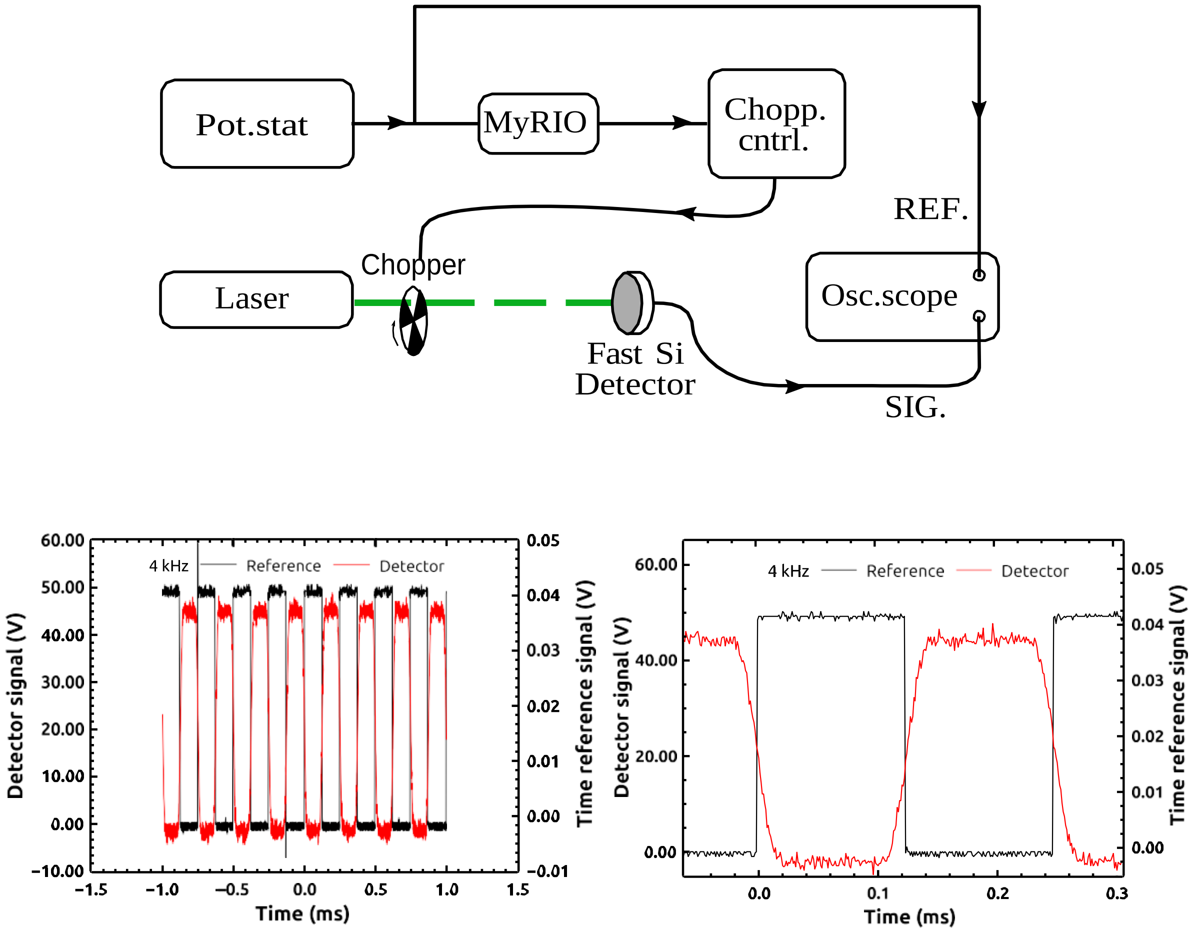

Figure S2: Top: signal flow for the synchronisation test on the top. Bottom: oscilloscope reading of the test performed at 4 kHz pumping frequency.

180 ° vs. 90 ° phase shift.

The delta Raman signal, is sensitive to changes in the average composition of the electrode. In the case of constant reaction rates during both the high and low crests of the SW, the 0/180° delta Raman, $\Delta R^{H}_{ref}/\Delta R^{L}_{ref}$, might not be informative since the average composition of the 0° phase signal would be equal to the 180° phase signal, see Fig. S3. In these cases delta spectra recorded at ±90° are the most descriptive since they probe the electrode when its average composition differs the most, at the edges of the SW.

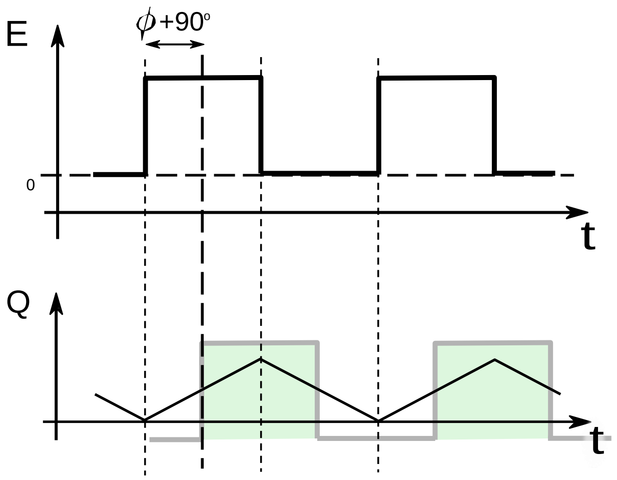

Figure S3: 90° phase shift case. In the top the potential SW, in the bottom the amount of species present at the electrode (Q, integrated current). In background (pale green) the Raman pulses.

RuN_6 electrochemistry

Even though the electrochemical behaviour of the RuN_6 complex is known from the literature, fast electrochemistry measurements have been repeated as described in the main paper.

The complex was grafted on the surface of a Pt microelectrode (100 μm diameter) by 8-amino-thiol linkers (via amide condensation, see main paper).The electrode was loaded in a three electrode cell and 100 mM $TBAPF_6$ in dried acetonitrile was used as supporting electrolyte. The electrolyte was acidified by a drop of trifluoro-acetic-acid. A fast ADC module was used to perform the IR compensation during fast cyclic voltammetry. The scan rate was changed from 1 V/s to 10 kV/s to resolve the isomerization reaction. Results are reported in Fig. S4, potential is plotted vs. Ag/Ag^+ (10 mM $AgNO_3$).

It can be seen that the cyclic voltammetry at low scan rates (1 V/s) shows only two redox waves, the oxidation of the RuN_6 complex, at ca. 0.95 V (vs. ref) and the reduction of the $RuON_5^+$ isomer, at 0.35 V. When the scan rate is increased to 10 V/s the isomer RuN_6^+ starts to be visible in the reduction at ca. 0.85 V. The isomer $RuON_5$ can also be seen as oxidation wave at around 100-200 V/s and it slowly disappear at very high scan rates (over 2 kV/s) due to complete reversibility of the RuN_6^+/RuN_6 reaction.

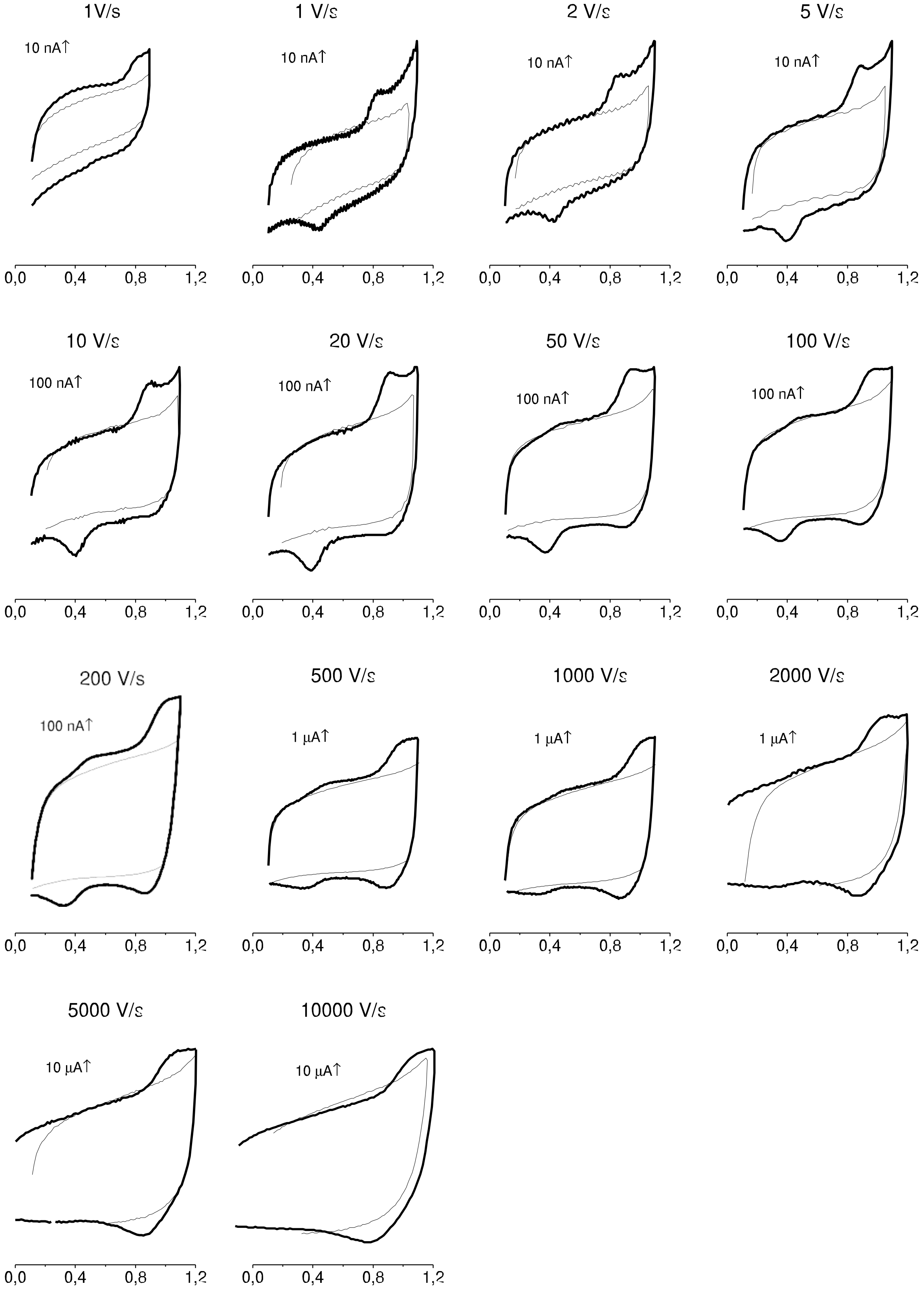

Figure S4: Cyclic voltammetry at various scan rates of the RuN_6 complex grafted on a flat Pt microelectrode surface by amino thiol linkers. Background currents (only amino-thiol grafted on electrode) are reported in thin lines.

The fitting of the peak current for the RuN_6^+/RuN_6 reaction confirms that the rate constant of this reaction is about 170 s^{-1}.

The peak currents ratios of the RuN_6^+/RuN_6 redox process were fitted to estimate the kinetics of the reaction and compare with the literature reported values. The results are repeated in Fig. S5.

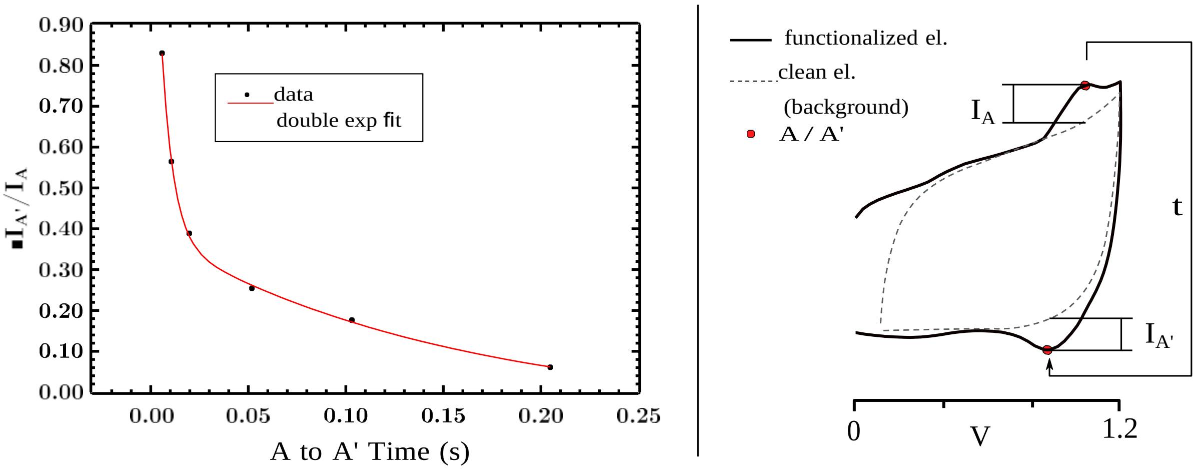

Figure S5: Current fitting of the RuN_6^+/RuN_6 redox peaks, taken after subtraction with background current. The expoenetial fitting resulted in 167 s^{-1} (ampl. 0.71) and 6.7 s^{-1} (ampl. 0.29). In the right figure, a typical sample CV with the indication of the peak current in respect to the background current.

Powder Raman spectra

A Raman spectrum of the RuN_6 complex recorded on a dry sample (crystals), using a 532 nm laser is reported in Fig. S6.

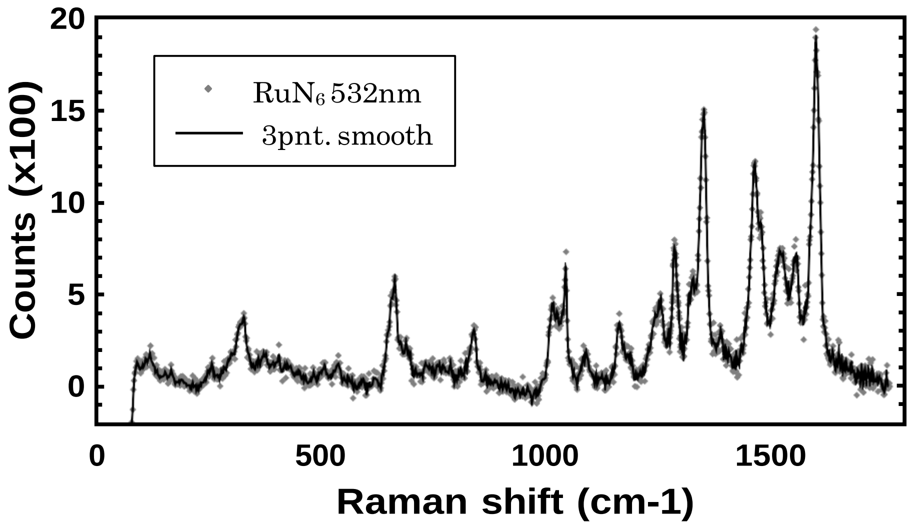

Figure S6: Raman spectrum of RuN_6 species in powder form. 532 nm laser, 10 mW power, 25 s total acquision time. The solid line is a 3 point smoothing of the data, which are reported as dots.

Raman imaging to locate/quantify hotspots.

The size (FWHM) of the Raman beam at its focal point can range from 2 to 50 μm, depending on settings, which can be significantly smaller than the size of the electrode (100 μm). To maximize the advantage of the SERS medium, imaging of the RuN_6 functionalized electrode was performed prior to the ERI measurements to test the possible heterogeneity of the amplification and to locate areas of high enhancement. The 2 μm focus point of the 785 nm laser (photon energy resonant with AuNP) was scanned step-wise on a round area of 100 μm diameter centered on the electrode. Raman spectra were recorded every 2 μm in the X and Y directions. A total of 7034 spectra were collected, two of which are plotted in Fig. S7. The black spectrum (magnified five times) is a typical not-amplified spectrum, reasonably due to a low surface concentration of AuNP able to enhance the Raman signal. Contrarily, the blue spectrum is a typical amplified spectrum showing bands in the 700-1600 cm^{-1} range, roughly in the same region as the dry sample spectrum in Fig. S6. Every collected spectrum was fitted with a 5th grade polynomial, producing the results reported in dashed lines in Fig. S7. Due to the structured vs.

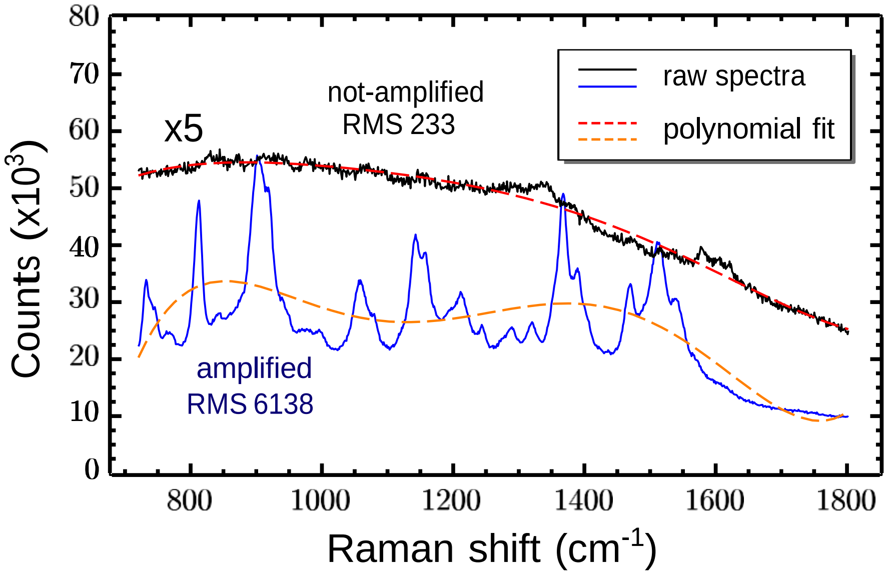

Figure S7: Raman spectrum of amplified vs. not-amplified RuN_6 fictionalised AuNP electrode (785 nm laser, 50 mW power, 2 s acquisition time). The counts of the not enhanced spectrum were multiplied by 5 times for clarity. RMS error of the respective fitting are reported too.

unstructured nature of the two kind of spectra, the fit of the not-amplified spectrum is quite good, in terms of error, compared to the one of the amplified spectrum. The root-mean-square (RMS) error of each fit was then used to evaluate the surface distribution of the AuNP, thus the surface heterogeneity of the Raman enhancement hot-spots. The RMS is plotted vs. the position of the collected spectrum in a 3D surface plot, reported in Fig. S8. In the same figure, the RMS analysis was also rendered as a gray-scale image, i.e. a top view of the electrode enhancement profile.

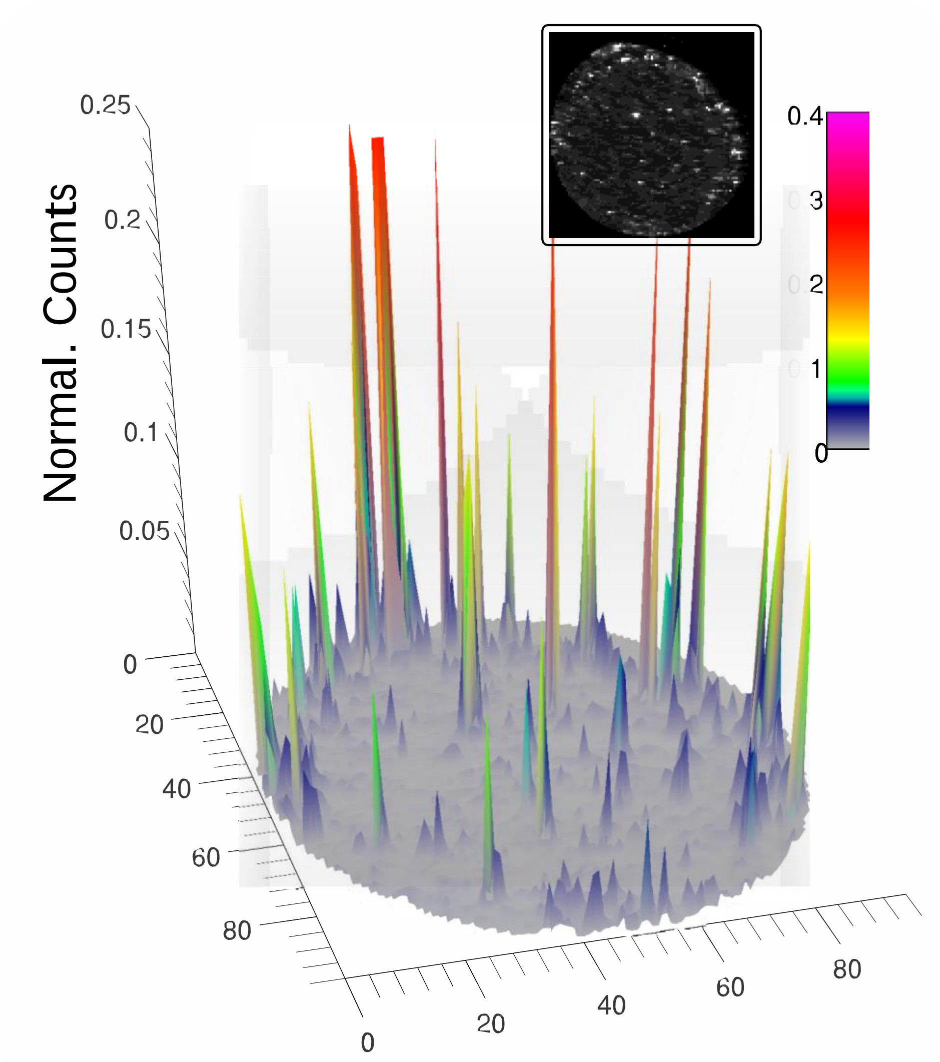

Figure S8: 3D rendering of the Raman imaging of the electrode surface created as described in the text; in the 2D inset the same set of data represented in a grey scale image. Experimental details: 7034 points, 785 nm laser, 50 mW power, 2 s acquisition time for each point.

From both, the 3D surface and the gray-scale image, it is possible to see that the

hotspots are more localized at the edge of the disk electrode. This is probably due to the electrochemical synthesis of the AuNP, accumulating the nanoparticles at the boundary of the electrode for edge effect of the electric field. From the 3D surface one can see that the amplified spectra are quite isolated events that occur in one or maximum two close image points. This indicates that the surface density of AuNP is not so high or not enough to produce a homogeneous enhancement. Clearly this can be a problem for the reproducibility of the collected EIR spectra, since the amplitude and structure depends on the position. The best solution found here was to trade enhancement with reliability. We have used a 15% defocused beam (*vide infra*), which produces a larger beam size at the focal point using the same objective, thus probing a larger area of the electrode. The defocusing is realized moving one of the lenses of the telescope of the spectrometer that is used to enlarge the laser beam, before the objective; the percentage of defocusing is taken as the percentage of focus distance of the lens.

Computational spectra

In the following, the intensities of the modes calculated by DFT are reported. A scaling factor of 0.9777 was used respect to the calculated values, as suggested in literature for the B3LYP functional with DZP basis sets.[?] The spectra shown in Fig. 7 are calculated from these intensities and frequencies used as parameters for a sum of lorenzian functions (with 15 cm^{-1} FWHM).

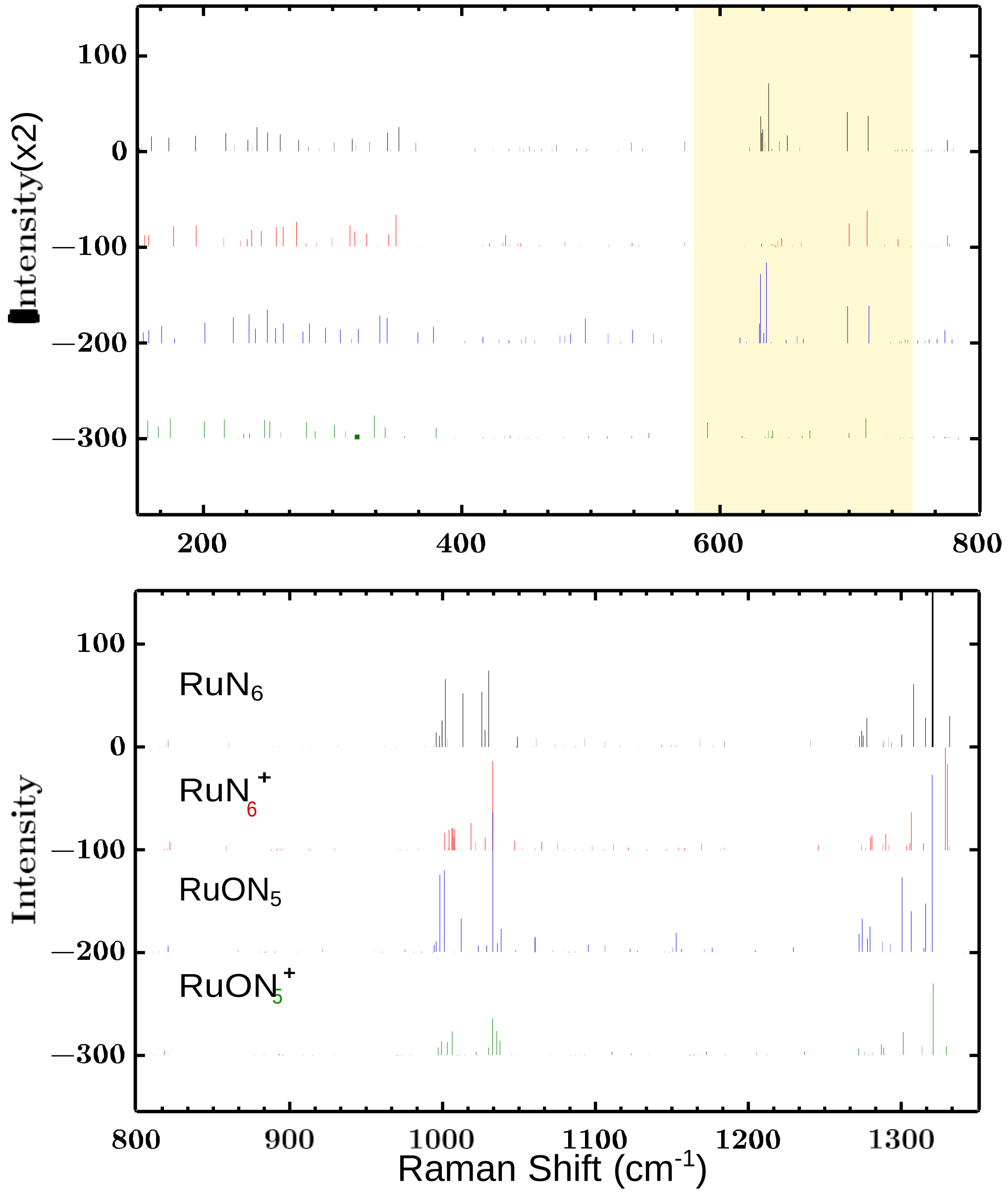

Figure S9: Vibrational Raman modes of the RuN6 family calculated by DFT. Intensities are given by simulating a spectrum scattered from a 785 nm laser. The intensities in the region 150- 800 cm1 were doubled for clarity. The spectral range of main interest has been highlited.

Control spectra

H/D isotope substitution.

The steady state spectrum of the RuN_6 complex in powder form has been collected after H/D exchange to help peak assignment. Powder of RuN_6 has been exposed to trifluoro acetic acid (TFA) to make sure the OH group was protonated, then Raman spectra were collected focusing on the powder crystals. The same procedure was repeated using deuterated TFA. The spectra are reported in Fig. S10, together with the calculated vibrational modes and intensities for comparison.

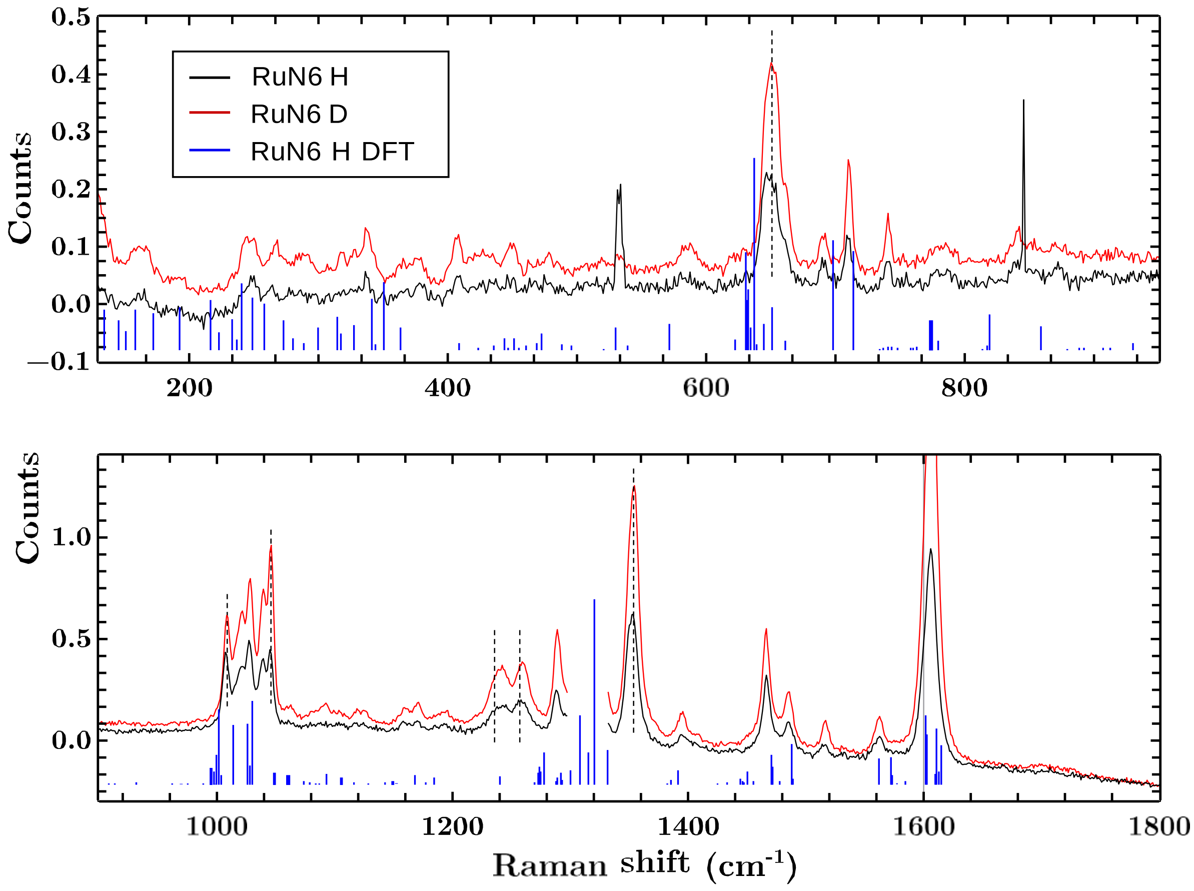

Figure S10: Raman spectra RuN_6 powder collected after exposure to trifluoroacetic acid (TFA) and deuterated trifluoro acetic acid (TFAD).785 nm laser, 10 mW power, 25 s total acquisition time was used.

In Fig. S10 the visible shifts are highlighted by dotted lines to facilitate the comparison. The shifting bands are supposed to be those related to the OH vibration, the same

one that should shift after the Ru-N/O isomerization. Indeed the frequencies selected here, 650, 1000-1050 and 1240 cm^{-1}, are also seen to be the most important ones in the EIR delta spectra, see main paper.

Amino-thiol linkers spectrum.

A steady state Raman spectrum of the AuNP loaded with only the 8-aminothiol and 6-exanthiol is reported in Fig. S11 for comparison with the full complex spectra.

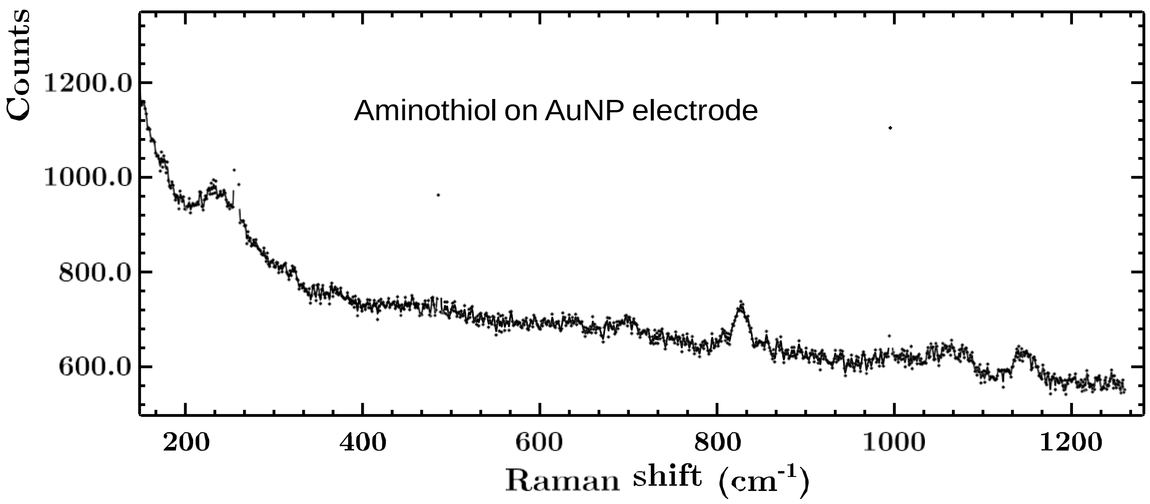

Figure S11: Raman spectrum of 8-aminothiol grafted on AuNP (785 nm laser, 10 mW power, 25s total average time). Cosmic ray spikes are removed.

Raw EIR spectra

Complex in solution.

Steady state *in-situ* Raman spectroscopy was performed with a solution of RuN_6 (5 mM, $TBAPF_6$ 100 mM as supporting electrolyte), using the same cell as reported in the main paper but with a flat Pt microelectrode (100 μm diam.). The complex was not grafted on the electrode but dissolved in solution. The 473 nm laser was used in this measurement to exploit the resonance enhancement effect of the MLCT (metal-to-ligand-charge-transfer) band of the complex, absorbing around 460 nm. Two spectra are reported in Fig. S12, one collected applying 0 V_{ref}, in blue, and 2 V_{ref}, in red. The laser was focused ca. 5 μm from the surface of the electrode, to maximize the probed solution volume. The spectrum of a solution of acetonitrile with the supporting electrolyte (with no complex) is reported in black. In this last case the laser power was 10 times (100 mW) the one used in the measurement with the complex.

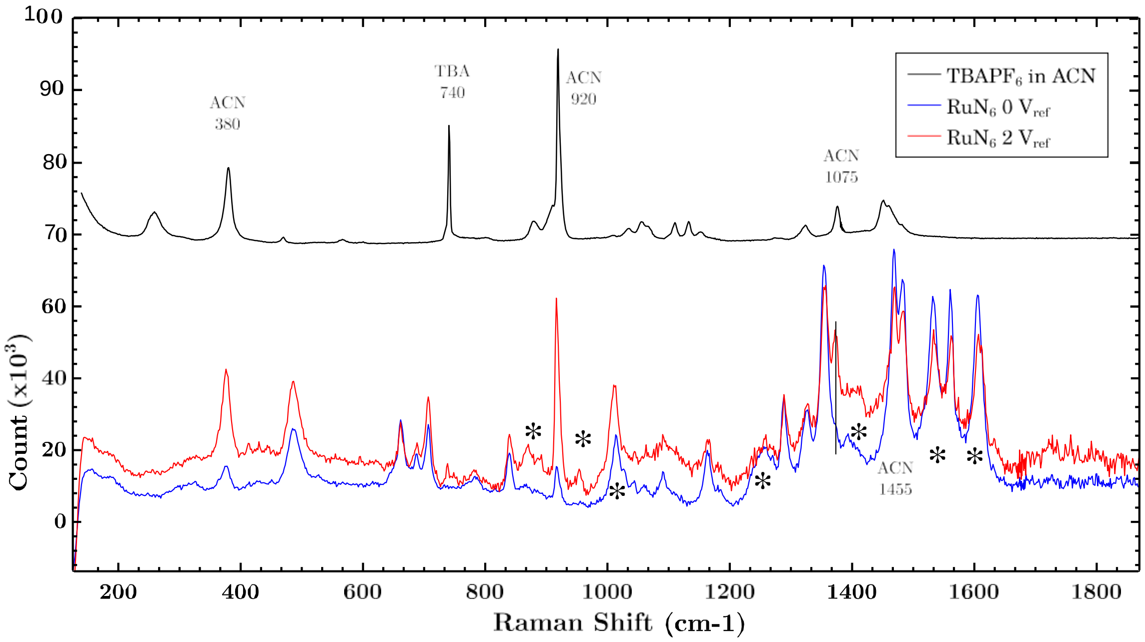

Figure S12: Raman spectra of the RuN_6 solution after application of 0 V_{ref}, in blue; 2 V_{ref}, in red; and the solution of $TBAPF_6$ in acetonitrile. 473 nm laser, 10 mW power for blue and red, 100 mW for black; 10 s total acquisition time.

In Fig. S12 both 0 V_{ref} and 2 V_{ref} show some peaks of the solvent or the electrolyte:

380, 740, 920, 1075 cm^{-1}. The most important differences between the two ***in-situ*** steady state spectra can be seen in the peaks at around 880, 950, 1010, 1240, 1400 cm^{-1} and the group around 1500-1600 cm^{-1}. These peaks are also observed in the EIR spectra at low and high SW frequencies (see main paper). This helps in the interpretation of the spectra in the search for the signal of transient species. Namely, in the difference peaks observed here, since they belong to a steady state measurements they should be regarded as exclusively belonging to the two stable species: RuN_6 and $RuON_5^+$.

Complex grafted on electrode.

In the following the raw spectra recorded at various pump SW frequencies (reported in the figures).

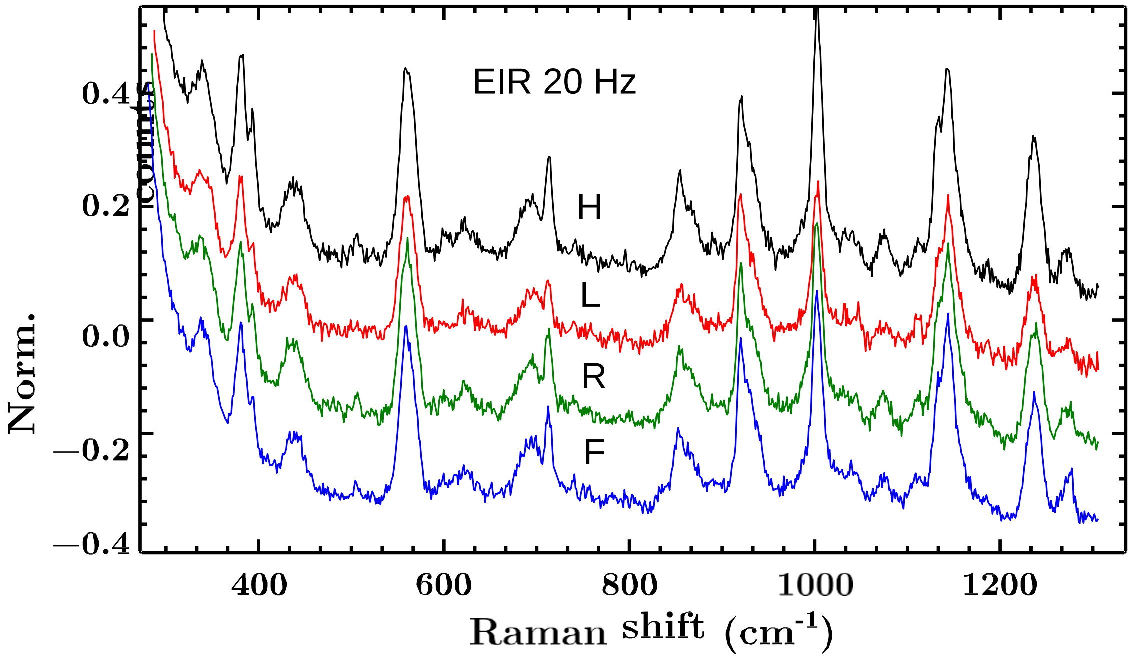

Figure S13: EIR spectra recorded at 20 Hz pump frequency at four different phase shift.

No steady state spectra (under constant bias) could be recorded due to the degradation of the Au nanoparticles. The oxidation potential of the RuN_6 is ca. 200 mV close to the oxidation potential of Au, which we experienced to be enough to initiate the desorption of the complex from the Au NP leading to a substantial decrease of the complex Raman

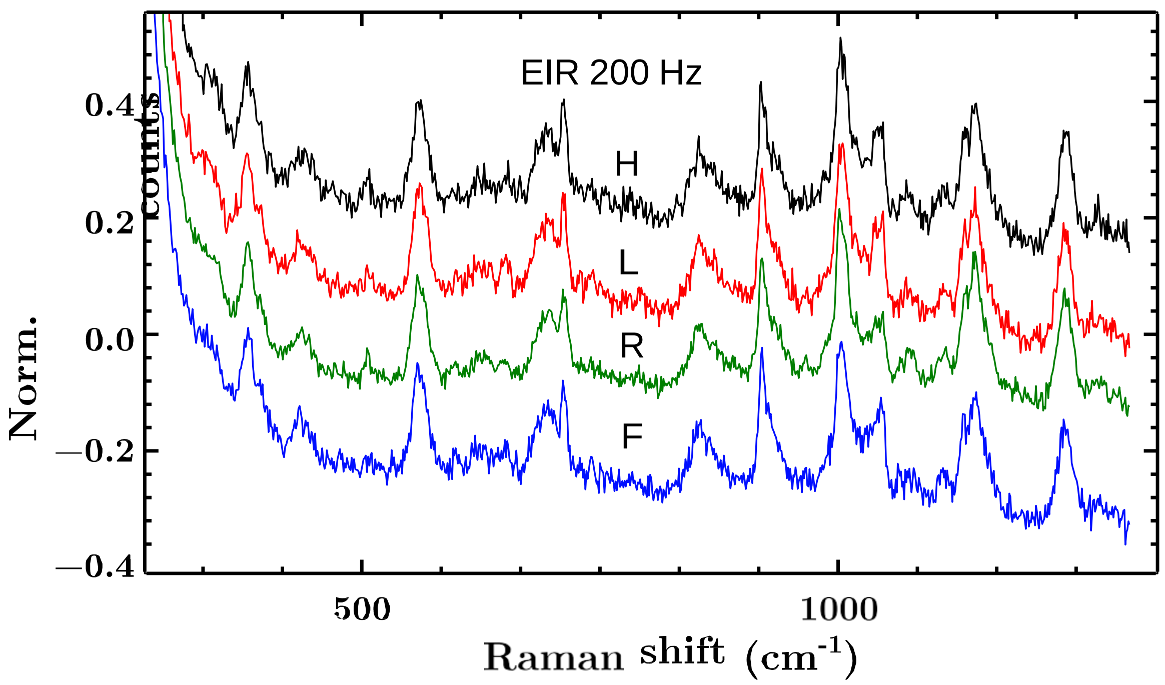

Figure S14: EIR spectra recorded at 200 Hz pump frequency at four different phase shift.

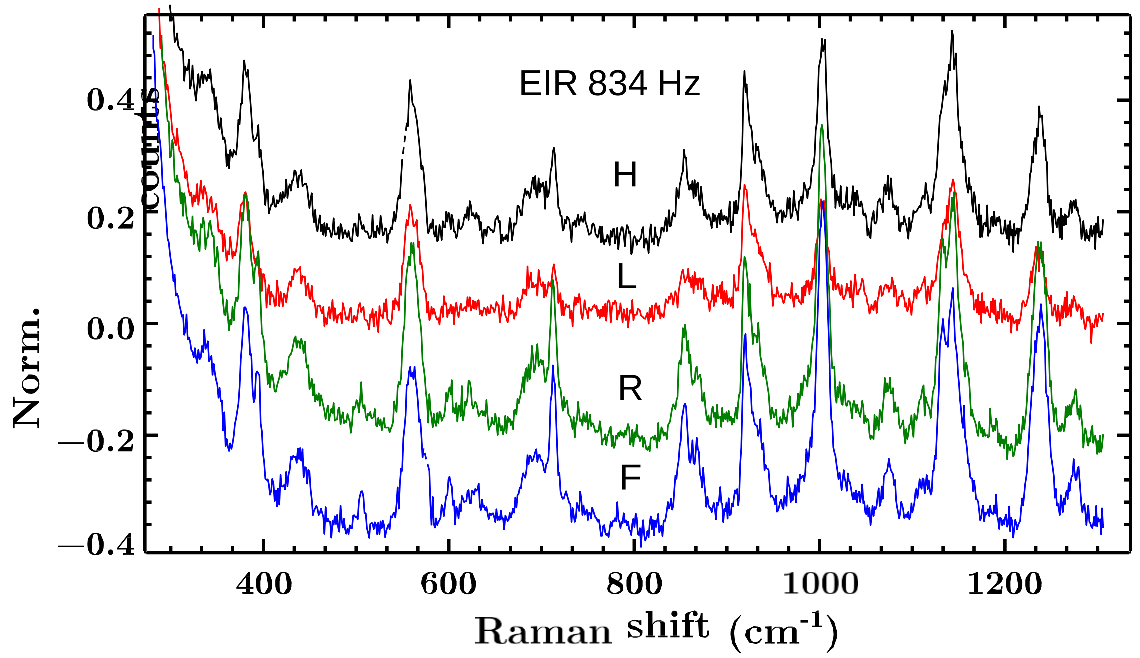

Figure S15: EIR spectra recorded at 834 Hz pump frequency at four different phase shift.

signal. Luckily desorption does not occur during SW potential cycling which enables EIR measurements. The spectra reported in Fig. S16 were collected at 20^{H}, 834^{H} and at open circuit potential (ocp). Interesting peaks are labelled with an asterisks for clarity. In the ocp spectrum the peaks at 650, 712, 1045 and 1290 cm^{-1} show lower or zero magnitude in the two EIR spectra in favour of new/larger peaks at: 340, 392, 437, 600, 712, 1075 and 1275 cm^{-1}.

Spectral assignment is done by comparison with computed (DFT) normal modes and via isotope labeling. The computed spectra of all four species are reported in Fig. 7

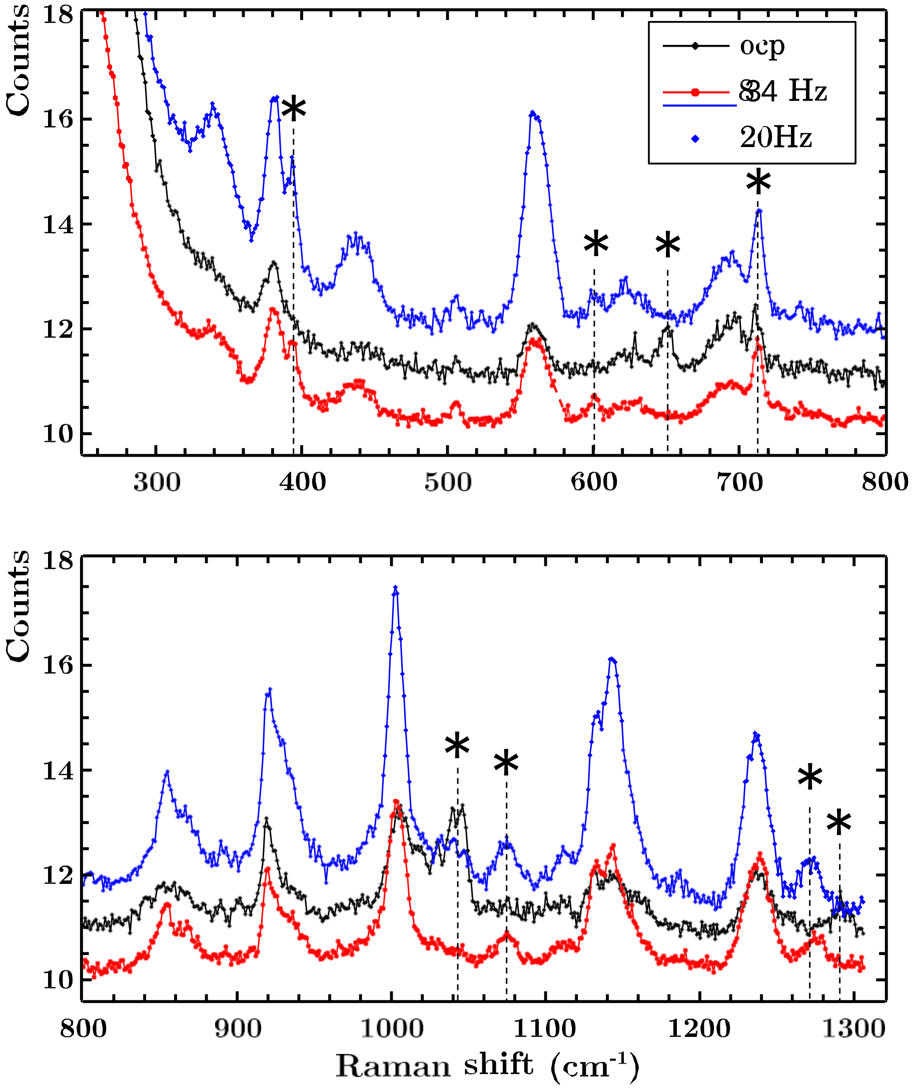

Figure S16: Raw EIR spectra of RuN_6 fuctionalized AuNP electrode at ocp, $20^{H}$and 834^{H}Hz. Cosmic rays are removed.

and Fig. S9.

A general rough assignment can be done as follows. The bands at low frequencies, 340, 392 cm^{-1}, are assigned to whole ring displacement (respect to Ru). These are affected by Ru- O/N isomerization and, to a smalle extent, by the Ru oxidation state. The bands at 650 and 712 cm^{-1} are assigned to ring stretching modes (comprehending the three Ru-N stretching bonds) of the BpyPyEt ligand in the RuN_6 isomer. The band at 600 cm^{-1} in the transient spectra, is instead assigned to a similar mode of the $RuON_5^+$ complex. At around 1000 cm^{-1} and 1300-1600 cm^{-1} one can find the C-H in plane bending and ring C-C stretching, respectively. Acetonitrile has two prominent peaks at 380 cm^{-1} and 920 cm^{-1}, the TBA salt shows only a relevant peak at 740 cm^{-1}, which was not observed here.

Detailed discussion of spectra.

From the electrochemical kinetics analysis is known that the $RuN_6^+ \longrightarrow RuON_5^+$ isomerization occurs with a time constant of 150-200 s^{-1} while the $RuON_5 \longrightarrow RuN_6$ is quite faster (500-1000 s^{-1}). Considering that the EIR spectra collected here reached a 0.6 ms semiperiod, we should be able to observe, at least, the RuN_6^+ isomer.

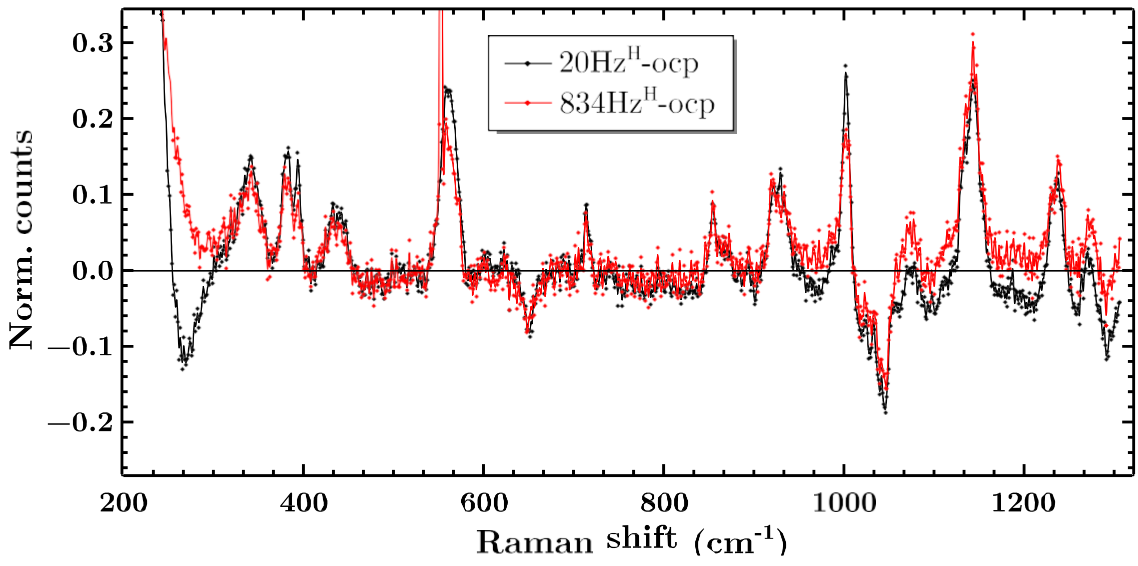

Figure S17: Delta EIR spectra of the RuN_6 functionalised AuNP electrode of normalized 20^H-ocp and 834^H-ocp.

To be able to compare the data from different species, delta spectra have been calculated. The raw spectra reported in Fig. S16 have been treated according to literature protocols to account for a different magnitude of enhancement. Briefly,the flat background B is subtracted from the raw spectra R and is also used to normalize them, the final spectra being S = (R-B)/B. The spectrum recorded at ocp has been taken as reference and subtracted to the 20^H and 834^H spectra producing the results reported in Fig. S17. The two delta spectra look quite similar except for a few bands, showing both positive and negative features. The 20^H shows negative bands at 250, 650, 1020-1060 cm^{-1} and around 1280 cm^{-1}; while it shows relevant positive bands at 340, 392, 712, 850, 1000, 1080, 1280 cm^{-1}.

It seems that the two delta spectra even though recorded at a quite different pump frequency, 20 and 834 Hz, must belong to similar chemical composition; this can be explained by the fact that the Raman probe is not a perfect square wave having rise/fall component of few angular degree (see Fig. S2) that when probing the High/Low state of the pump might miss the first and most important part of the transient signal. Indeed, as mentioned in the description of the technique, in EIR the full information regarding a time point in the time evolution is given by collecting and analysing all four phases spectra. The delta spectra, collected at 843 Hz at all four the phase shifts, are reported in Fig. S18.

Here it is clear that some positive peaks can be seen in the +/-90° (R and F, green and blue respectively), that are not present or are not as strong in the 0/180° (L and H, black and red). The 834^L-ocp spectrum is the one that should most resemble the ocp spectrum, since it probes the low state of the pump SW. Indeed, the 834^L-ocp delta spectrum shows the least number of features.

It should be emphasized that the delta spectra collected with EIR, should be interpreted considering that the probed state (H vs. L) is a result of the preceding pump state (L vs. H, respectively). In other words, every spectrum at a specific phase shift, H/L/R/F, contains information of the other phases too. At fast SW pump frequencies, where the unstable species should be observable, considering that the $RuON_5 \longrightarrow RuN_6$

isomerization is estimated to be quite faster than the $RuN_6^+ \longrightarrow RuON_5^+$ (respectively, >500 s^{-1} and 130 s^{-1}), the chances to detect the $RuON_5$ isomer in the L or F spectra are lower than the respective RuN_6^+ in the H or R spectra.

This means that the 834^L spectrum is not perfectly resembling the ocp spectrum but most likely contains also features of the High state of the pump SW, i.e. the 834^L-ocp delta spectrum is not a zero line but it shows positive and negative features too. This is not an entirely negative point since it helps in the interpretation of the other delta spectra. From this observation it is clear that the delta peaks at 350, 380, 440, 560, 940 and 1040 cm^{-1} belong to the High state of the SW which can then be assigned to the $RuON_5^+$. This is confirmed by the slightly larger magnitude of some of these peaks in the 834^H-ocp delta spectrum and from comparison with the simulated spectra. Indeed one can observe that in the computed $RuON_5^+$, in Fig. S9, there are bands at around 340 and 380 cm^{-1} that are absent in the RuN_6 spectrum. The absence of negative features in the 834^L-ocp can be attributed to the similarity of the RuN_6 and $RuON_5^+$ spectra in the low frequency region. One more confirmation of this assignment is the omnipresent negative band at 1050 cm^{-1} which can be assigned to the strong modes at 1013-1030 cm^{-1} of RuN_6, see Fig. S9, which are absent (or largely reduced) in the simulated spectra of all the other species. The negative peak at 650 cm^{-1} is also present in all the spectra.

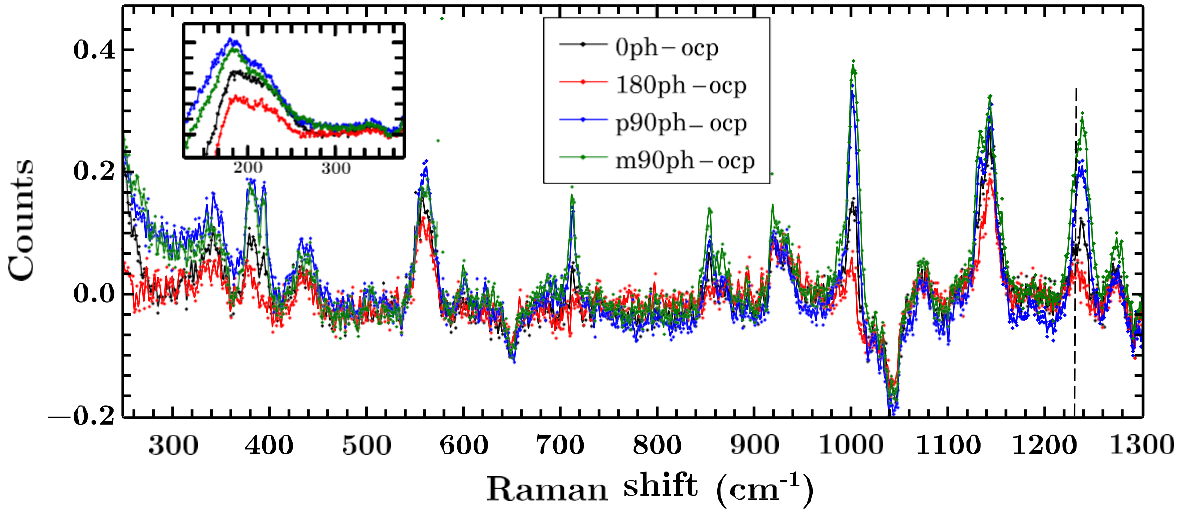

Figure S18: EIR spectra recorded at 834 Hz pump frequency at four different phase shift.

The assignment of this peak can be done, by comparison with computational data, to the modes at around 635 cm^{-1} of the RuN_6 species, i.e. to the ring deformation lead by the N-Ru-N symmetric stretching of the BpyPyEt ligand. This mode is absent in the Ru(III) isomers but it is present (with a small shift) in the $RuON_5$ isomer. As shown earlier, it is reasonable to assume that the $RuON_5$ species would have a small contribution in these spectra, which can explain why the 650 cm^{-1} negative band is present in all delta spectra. Now the attention should be focused on the positive features of the 834-ocp delta spectra, 392, 600, 712, 1002, 1132, 1238 cm^{-1}. The peak at 392 cm^{-1} is visible only on the H phase spectra and it becomes more prominent in the R/F phase spectra, together with a peak at around 385 cm^{-1} that seems to be significantly shifted from the acetonitrile peak at 380 cm^{-1}(see vertical dotted line in Fig. S18). The peak at 392 cm^{-1} can be assigned to the $RuON_5$/$RuON_5^+$ species which present vibrational modes in this region that are missing in the RuN_6/RuN_6^+ isomers. The shifted acetonitrile peak observable in the H spectrum and with an increased intensity in the R and F spectra could indicate a participation of the solvent in the isomerization reaction. Indeed, computationally it was observed that a solvent molecule is beneficial to the stabilisation of the hydrogen in the unbound ligand. Further speculation in this direction is not possible with the present set of data and beyond the scope of this work.

The peaks at 600 and 712 cm^{-1} are perhaps the most significant regarding the probe of transient species. The computational spectra show that the stable species RuN_6 has multiple modes in the region 620-670 cm^{-1}, see Fig. S9, indeed showing as a broad band at about 650 cm^{-1} in the ocp spectrum, see Fig. S16; RuN_6 also shows two peaks at 698 and 715 cm^{-1}. The simulation for the stable oxidized isomer $RuON_5^+$ does show a new (weak) peak at 590 cm^{-1}, does not show relevant peaks in the 620-670 region and shows only a not so strong mode at 713 cm^{-1}. On the contrary the calculated unstable species RuN_6^+ shows a strong peak at 714 cm^{-1} and very weak peaks in the 620-670 cm^{-1} region. Also the transient $RuON_5$ species that has strong peaks at 698 and 715 cm^{-1} and two modes around 630 cm^{-1}. A similar behaviour is observed in the experimental EIR spectra. In Fig. S18 a positive 600 cm^{-1} peak is observed in the R and F phase delta

spectra together with a broad negative band at around 650 cm^{-1}; moreover, a sharp positive feature absent in the L delta spectrum, is present with medium intensity in the H spectrum but quite prominent in the R and F delta spectra. Overall this indicates the origin of the 712 cm^{-1} peak to transient species. The 712 cm^{-1} peak is present in both the unstable isomers RuN_6^+ and $RuON_5$ in the calculated and experimental spectra, thus it cannot be used to differentiate between these two species. In any case, they are both transient intermediates visible only at sub-ms timescales, proving the effectiveness of the EIR technique.

Few important peaks can be found in the 800-1300 cm^{-1} region. The most prominent is the positive peak at 1005 cm^{-1}which can be related to the negative peak at 1050 cm^{-1} and the positive one at 1075 cm^{-1}. From the computed spectra the negative band can be associated with a strong RuN_6 mode at 1013 cm^{-1}, which is absent in all the other Ru species. Instead, the amplitudes of the modes at around 1000 cm^{-1} and 1050 cm^{-1} are weaker in the $RuON_5^+$ isomer but stronger in the transient RuN_6^+ and $RuON_5$ species. This again confirms that the peaks prominent at R and F phase shift are more related to the transient species. The peaks at 858, 1130 and 1238 cm^{-1} can all be assigned to modes in the computational spectra even though they are all of weak magnitude, while they appear of relevant intensity in the EIR spectra.

As discussed in the main paper, the peaks at 858, 1130 and 1238 cm^{-1} can all be assigned to modes in the computational spectra even though they are all of weak magnitude, while they appear of relevant intensity in the EIR spectra. In particular the peak at 1238 cm^{-1} could be potentially used to differentiate all four species. According to calculations this mode is associated to either the C-COH-C symmetric stretching in the Ru-N isomers or the O-H-N stretch in the Ru-O species; interestingly this peak shifts about 10 cm^{-1} in the same isomer when oxidized/reduced and about 20 cm^{-1} due to the isomerization. In Fig. S18 a dotted line is added to help estimate the frequency shift. As the calculation predicts, a shift of ca. 20 cm^{-1} is found between the peaks assignable to the two stable species, i.e. in the H and L spectra, while the shift is about 5 cm^{-1} within the same isomer couple.

Kinetic trace and EIR spectra simulations

In order to compare the experimental EIR spectra with the DFT calculated spectra, linear combinations of these last ones can be calculated to reproduce the spectra of each EIR measurement. The factor of the linear combination are simply the composition ratios of the isomers at each phase and pulse width of every EIR measurement. The computation of the composition ratios required simulations of the time dependent concentrations of the RuN_6 isomers during the application of the pumping SW. For the simulations few assumption were made: the electron transfer is instantaneous (in respect to the isomerization reactions, this is justified by the respective rate constants, see main paper); the isomerization reactions obey to a kinetics of the first order, i.e. evolve with an exponential law, of the form $[A]=[A]_0 \cdot e^{-t \cdot k}$). The estimation of the composition ratio is done by performing a weighted integration of the species kinetic traces created by the pump square wave pulses. The kinetic traces are defined by the initial concentration of the evolving species and the kinetic rate constant of the process. Considering the isomerization cycle reported on Fig. 5 of the main paper, and redefine for convenience as:

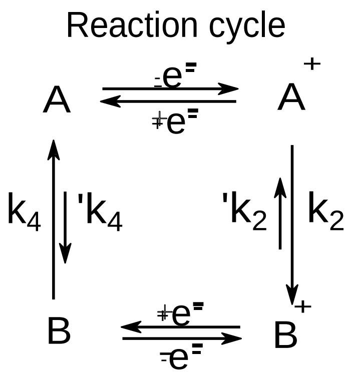

Figure S19: Reaction cycle used in the simulations corresponding to the cycle in Fig. 5. k_2 and k_4 are ca. 150-200 and 500-1000 s^{-1}, respectively (e^- transfers occur in ca. 10^4s).[S31]

The kinetic laws for each species can be defined accordingly. In the *low* state of the pumping SW, the A and B species can be described with the laws:

$$[A]_t = [A]_{nc} + [B]_0 \cdot (1 - e^{-t \cdot k_4})$$

$$[B]_t = [B]_0 \cdot (e^{-t \cdot k_4})$$

While in the ***high*** state of the SW the A^+ and the B^+ can be described with the laws:

$$[A^+]_t = [A^+]_0 \cdot (e^{-t \cdot k_2})$$

$$[B^+]_t = [B^+]_{nc} + [A^+]_0 \cdot (1 - e^{-t \cdot k_2})$$

Here it has been assumed that if an isomerization is not complete at the moment of the switching potential, e.g. the conversion of A^+ to B^+ at the ***high-to-low*** step, the ***non-converted*** A^+ will be instantly and fully converted in A, this quantity appears as $[A]_{nc}$ (and $[B^+]_{nc}$ for the ***low-to-high*** case). The initial concentration of the species, $[A^+]_0$ and $[B]_0$, are also depending on the pumping frequency. This is due to the fact that at low frequencies the isomerization reactions will be complete, thus the initial concentrations will be related to the whole amount of material, while at fast frequencies, when the isomerization reactions do not reach completeness some the initial concentration will be related to a part of the total amount. In other words counting as ***unity*** the whole active material then we find $[B^+]_{nc} + [A^+]_0 = 1$ and $[A]_{nc} + [B]_0 = 1$. The calculation of the initial concentrations $[A^+]_0$ and $[B]_0$ is done by iteration using the above equations, starting from $[A^+]_0 = 1$ and calculating the $[A]_t$ at the time t given by the width of the pumping pulse, then this concentration gives $[A]_{nc}$ which is used to calculate $[B]_0$, thus $[B]$ at time t, and so on up to convergence. A table of the initial concentrations calculated in this way is reported in Table S1. $k_2 = 200\ s^{-1}$ and $k_4 = 1000\ s^{-1}$ were used.

Table S1: Initial concentrations, $[A^+]_0$ and $[B]_0$, and ***non-converted*** concentrations, $[A]_{nc}$ and B^+_{nc}, expressed in fuction of pump pulse width.

Species \ Pulse width	0.5 s	0.05 s	0.005 s	0.0006 s
$[A^+]_0$	1	1	0.996	0.88
$[B^+]_{nc}$	0	0	0.0042	0.12
$[A]_{nc}$	0	$4.5e^{-05}$	0.37	0.78
$[B]_0$	1	0.99995	0.63	0.22

The final simulations are then performed by integrating the above equations for the kinetic laws with interval and weight function that emulates the pump pulse profile. The pump profile is not a perfect square, it is instead trapezoidal (this is due to the production of the pulses via chopping a finite sized laser beam with a 50-50 duty cycle), see Fig. S2. The integration weighting profiles are illustrated in Fig. S20 (in this cartoon the kinetic traces do not correspond to the real ones, they have been modifies for clarity purposes).

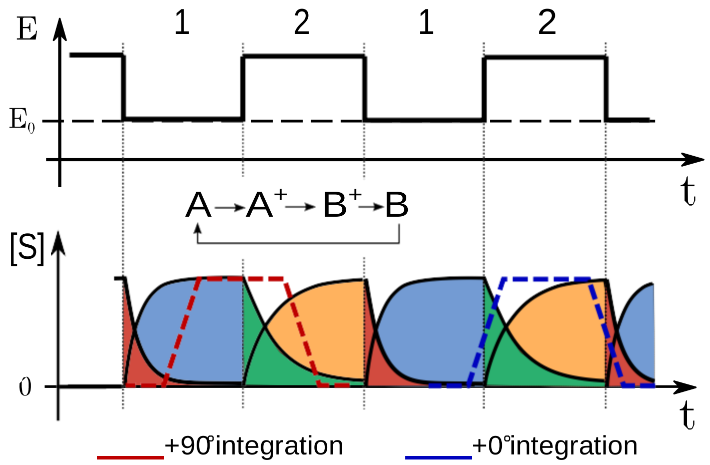

Figure S20: Dashed curves, weighting functions used in integration, examples shown for the $+90^{\circ}$ and 0° phase. Solid lines, example of concentration profile in a full SW cycle.

From Fig. S20 is clear how the trapezoidal shape introduces a mixing of the composition in the various pump states. For example, in the 0° integration (dashed blue) the main contribution will be given by the B^+ and A^+ species but there will be also some contribution from B and A. The mixing contribution is pump frequency dependent. In the real integration the same kinetic constant used for the calculation of the initial concentrations have been used (k_2 = 200 s^{-1} and k_4 = 1000 s^{-1}). The trapezoidal weighting function has been taken so that the rising and falling components are (together) the 10% of the pulse.

The results of the integration are reported in Table S2 and are given as function of pump pulse width and measuring phase. Since the scope of the simulation is to support the validity of the method to isolate the unstable isomers the table reports also the ratio A^+/B^+ which correspond to the ratio of the $RuN_6{}^+/RuON_5{}^+$species.

Table S2: Spectral component factors expressed in function of pump pulse width and measuring phase.

Species	Phase	0.5 s	0.05 s	0.005 s	0.0006 s
A	H	0.024	0.019	0.018	0.021
	L	0.97	0.96	0.86	0.81
	R	0.50	0.50	0.49	0.43
	F	0.50	0.48	0.38	0.40
B	H	0.00096	0.0063	0.0074	0.0043
	L	0.0010	0.014	0.12	0.16
	R	~0	~0	0.0097	0.071
	F	0.0020	0.020	0.12	0.095
A^+	H	0.0060	0.089	0.61	0.81
	L	0.0040	0.011	0.017	0.021
	R	0.010	0.099	0.39	0.43
	F	~0	0.00070	0.24	0.40
B^+	H	0.97	0.89	0.36	0.17
	L	0.021	0.014	0.0081	0.0043
	R	0.49	0.40	0.11	0.073
	F	0.50	0.50	0.26	0.098
$\frac{A^+}{B^+}$	H	0.0062	0.10	1.7	4.8
	L	0.19	0.74	2.1	4.9
	R	0.020	0.25	3.6	5.8
	F	~0	0.0014	0.91	4.1

It can be seen that the ratio A^+/B^+ is maximized with shortest pump pulse width and in the R measuring phase, as it was expected. It should be also noted that in the R phase the absolute signal of the A^+ species is about the half (0.43) the one obtained in the H phase (0.81) in the same pump pulse width. This is due to the fact that half of the signal in the R phase comes also from the L phase, which contributes mainly with a signal from A (0.35). This is not a problem since the EIR spectra are represented in a difference form where the raw spectra are subtracted and normalized vs. a reference spectrum which, in

this case, is the RuN_6 spectrum, i.e. the *A* species. This has the effect of enhancing the signal from the H component in the R spectrum, which is beneficial for our scopes.

The values just calculated have been used, as weighting factors, to simulate the EIR spectra by a linear combination of the DTF computed spectra (shown in Fig. 7 of the main paper). The results for slowest (500 ms) and fastest (0.6 ms) pump pulse width are reported in Fig. S21.

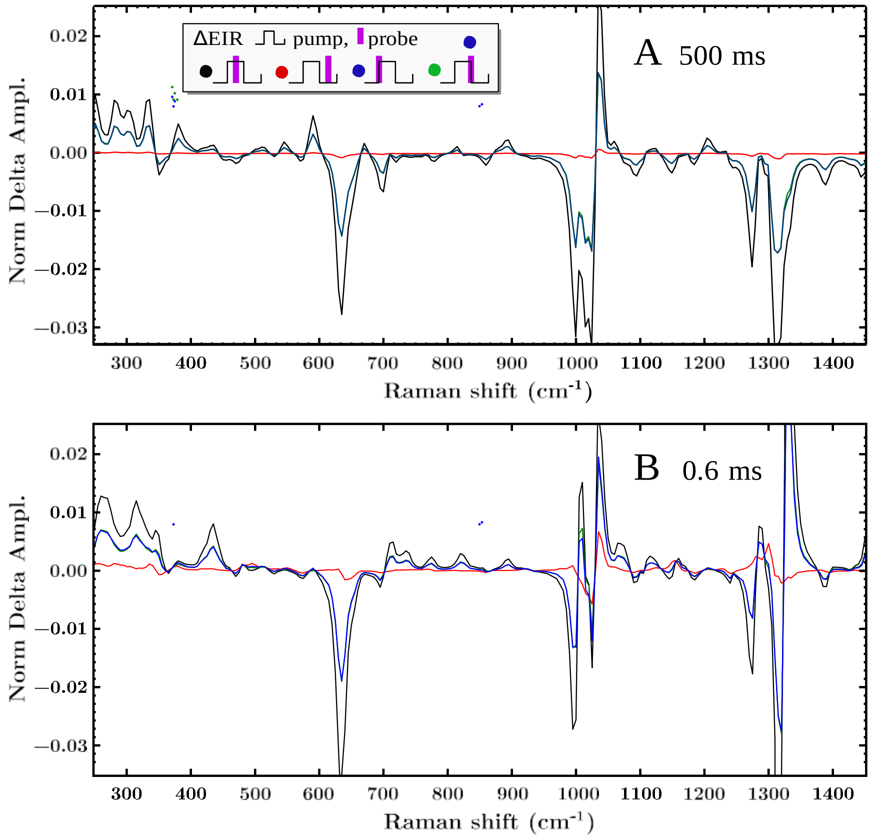

Figure S21: Simulated ΔEIR spectra of the (A) 500 and (B) 0.6 ms pulse width measurements. The species RuN_6 has been taken as reference and subtracted to the simulates raw spectra to produce the difference spectrum.

It can be appreciated how the peak at around 700-750 cm^{-1}, which was taken, in the main paper, as a reference signal for the elusive isomer, changes in respect to the pump pulse width; it is basically absent in the 500 ms spectra and it is prominent in the 0.6 ms spectra. This confirms the experimental results described in the main paper.

In Fig. 9 in main paper, the normalized amplitude of the peak at 712 cm^{-1} is reported as function of the pump SW period (taken as a measure of time). The peaks amplitudes

were taken averaging the 5 data points at the peaks maximum of the R^H EIR spectra. An arbitrary pump period value of 10 s was given to the ocp data point. The simulated curve in Fig. 9 is an exponential decay function with time constant 200 s^{-1}, amplitude and offset so to match the ocp data value (1.1) at infinite times and the highest data value (2.2) at the shortest times.

Further material details

All solvents and chemicals, acetonitrile, $HAuCl_4$, 8-amino-octanthiol (8AT), 6-mercaptohexane (6MH), 4-(4,6-dimethoxy-1,3,5-triazin-2-yl)-4-methylmorpholinium chloride (DMTMM), tetrabuthylammonium hexafluorophosphate ($TBAPF_6$), trifluoro acetic acid and its deuterated form (TFA and TFAD), H_2SO_4 (99.999% purity), were bought from Sigma-Aldrich and used as received. The Ru-based molecular switch complex, was synthesized in house following a procedure published elsewhere.[S31] Au and Pt wires (99.999% purity, 50 and 100 μm diam.) to be used in microelectrode fabrication were purchased from Goodfellow.
